# Thermally Insulating, Thermal Shock Resistant Calcium Aluminate Phosphate Cement Composites for Reservoir Thermal Energy Storage

**DOI:** 10.3390/ma15186328

**Published:** 2022-09-12

**Authors:** Toshifumi Sugama, Tatiana Pyatina

**Affiliations:** Brookhaven National Laboratory, Upton, NY 11973-5000, USA

**Keywords:** lightweight cement, thermally insulating cement, reservoir thermal energy storage, geothermal cement, thermal-shock-resistant cement, hydrophobic cement, silica aerogel, calcium phosphate cement

## Abstract

This paper presents the use of hydrophobic silica aerogel (HSA) and hydrophilic fly ash cenosphere (FCS) aggregates for improvements in the thermal insulating and mechanical properties of 100- and 250 °C-autoclaved calcium aluminate phosphate (CaP) cement composites reinforced with micro-glass (MGF) and micro-carbon (MCF) fibers for deployment in medium- (100 °C) and high-temperature (250 °C) reservoir thermal energy storage systems. The following six factors were assessed: (1) Hydrothermal stability of HSA; (2) Pozzolanic activity of the two aggregates and MGF in an alkali cement environment; (3) CaP cement slurry heat release during hydration and chemical reactions; (4) Composite phase compositions and phase transitions; (5) Mechanical behavior; (6) Thermal shock (TS) resistance at temperature gradients of 150 and 225 °C. The results showed that hydrophobic trimethylsilyl groups in trimethylsiloxy-linked silica aerogel structure were susceptible to hydrothermal degradation at 250 °C. This degradation was followed by pozzolanic reactions (PR) of HSA, its dissolution, and the formation of a porous microstructure that caused a major loss in the compressive strength of the composites at 250 °C. The pozzolanic activities of FCS and MGF were moderate, and they offered improved interfacial bonding at cement-FCS and cement-MGF joints through a bridging effect by PR products. Despite the PR of MGF, both MGF and MCF played an essential role in minimizing the considerable losses in compressive strength, particularly in toughness, engendered by incorporating weak HSA. As a result, a FCS/HSA ratio of 90/10 in the CaP composite system was identified as the most effective hybrid insulating aggregate composition, with a persistent compressive strength of more than 7 MPa after three TS tests at a 150 °C temperature gradient. This composite displayed thermal conductivity of 0.28 and 0.35 W/mK after TS with 225 and 150 °C thermal gradients, respectively. These values, below the TC of water (TC water = 0.6 W/mK), were measured under water-saturated conditions for applications in underground reservoirs. However, considering the hydrothermal disintegration of HSA at 250 °C, these CaP composites have potential applications for use in thermally insulating, thermal shock-resistant well cement in a mid-temperature range (100 to 175 °C) reservoir thermal energy storage system.

## 1. Introduction

This study is a continuation of efforts in designing cement composites with very low thermal conductivity (TC) under water-saturated conditions for use in reservoir energy storage and heat recovery wells [1]. For deep wells, significant energy savings are made possible if insulating cement is used for heat storage and recovery wells. The economic benefits of using insulating cement can amount to more than 75% heat loss reduction with a 50% decrease in cement thermal conductivity (TC) for a 3 km deep well [2]. Insulation can also decrease heat losses in borehole underground storage systems [3]. Research on thermally insulating cementitious materials for underground reservoirs is ongoing. Use of various lightweight particles including perlite, fly ash cenospheres, glass fibers, fumed silica, foam slurry technologies, and porous geopolymers have been reported [4,5,6,7,8]. These studies focused on relatively low-temperature (below 100 °C) applications and low thermal shock gradients. High-temperature reservoir thermal energy storage installations are among the set of solutions outlined in the Energy Storage Grand Challenge Roadmap by the U.S. Department of Energy. These solutions aim to accelerate the development, commercialization, and utilization of next-generation energy storage technologies [9]. Some hybrid installations are especially attractive because they are not limited to locations with existing geothermal resources and, as the heat is provided from an external source, the productivity of such resources does not decline over time [10].

In an earlier study, we reported the formulation and testing of a highly hydrophobic lightweight cement composite with a TC of 0.4–0.5 W/mK under water-saturated conditions [1]. Such low TC was achieved by using hollow fly ash cenospheres (FCS) treated with polymethylhydrosiloxane (PMHS) that chemically tailored the microsphere surfaces, making them hydrophobic and stable under alkaline cement environments.

In this work, we aimed to decrease the TC of insulating cement composites further, for applications in energy storage and recovery. One of the materials of interest—with a very high volumetric fraction of air—is hydrophobic silica aerogel (HSA). HSA has a ~99.8 vol.% porosity and a low density of 0.005 g/cm^3^, which results in its extremely low TC of 0.01–0.02 W/mK [11]. For a material with such low density, an important concern is its mechanical strength. Although very low TCs can be achieved using HSA, the mechanical properties of HSA-containing composites are generally poor. For instance, when using HSA with a 0.1–0.3 g/cm^3^ density and ~0.02 W/mK TC, the TC value of concrete samples containing 0, 40, and 60 vol.% aerogel, prepared in a water-saturated environment at room temperature, was ~1.8, ~0.9, and ~0.3 W/mK, respectively, with corresponding compressive strengths of ~8400 psi (~57.9 MPa), ~2900 psi (~20.0 MPa), and ~1200 psi (~8.3 MPa) [12]. Thus, a high compressive strength of aerogel-free concrete (~1.8 W/mK TC) with ~8400 psi (~57.9 MPa) compressive strength was reduced six-fold with 60 vol.% HSA.

One of the key factors for using HSA to provide good thermal insulation in water environments, is the application of a surface treatment to makes this material hydrophobic. A hydrophobic HSA can be prepared by substituting the hydrophilic silanol (-Si-OH) groups occupying the surface sites of silica aerogel with hydrophobic trimethylsilyl [-Si-(CH_3_)_3_] groups from hexamethyldisilazane (HMDS) [13,14,15,16]. The water absorption of hydrophilic aerogel is four to five times its weight compared with hydrophobic aerogel, which absorbs less than 2 times its weight water [17]. Another possible advantage of this treatment is improved hydrothermal stability. In the field of membrane molecular sieves, the hydrophobic surface preparation of mesoporous silica by trimethylsilyl groups led to two improved properties: reduction of water ingress into the porous structure and improved hydrothermal stability of membranes [18,19,20]. As the ≡Si-O-Si≡ linkage in hydrophilic silica is susceptible to alkali hydrolysis at around 60 °C (≡Si–O–Si≡ + H_2_O → 2 ≡Si–OH and ≡Si–O–Si≡ + OH^−^ → ≡Si–OH + ^−^O–Si≡), the hydrophobicity of organosilica improved the hydrothermal stability of porous silica at temperatures of 150 °C [21]. Therefore, porous HSA appears to have great potential for use in thermally insulating building material under atmospheric environments, including moderate moisture and vapor, and in hydrothermally stable molecular sieve membranes, at temperatures as high as 150 °C.

To improve the poor performance of ultralightweight HSA in developing cement with high mechanical strength, FCS, made of hard dense pozzolan shell that encapsulates CO_2_ and N_2_ as major and minor gases, respectively, can be used with HSA as a hybrid dual insulator. FCS are hollow aluminum-silicate (mostly mullite) particles collected as a byproduct at coal combustion plants [22]. The TC of FCS is 0.13 to 0.38 W/mK, bulk density is 0.5 g/cm^3^ [23,24], and compressive strength is ~22 MPa [25]. Unlike HMDS-treated HSA, FCS are subject to alkaline degradation in cement slurries at high hydrothermal temperatures. Such degradation in PR with precipitation of calcium-silicate-hydrate or calcium-aluminosilicate-hydrate gel reaction products [26,27,28] can lead to the damage of the shells, gas release, and problems with cement integrity.

E-glass and carbon microfibers can be used for the reinforcement of thermally insulating cements. A combination of two types of fibers was shown to improve tensile and flexural properties of epoxy composites [29,30,31]. In a damage and deflection study [32] of hybrid carbon/glass fiber polymer composites subjected to an impact load, it was observed that the carbon fiber surface in conjunction with the underlying glass fiber core acted to minimize the impact damage. In contrast, the surface layer of glass fibers alone suffered severe damage, resulting in localized delamination. These results strongly demonstrate that the high rigidity and stiffness of carbon fibers offer great resistance to impact damage, compared with glass fibers alone. Furthermore, E-glass fibers displayed far better thermal insulating performance than carbon fibers. The TC of unidirectional glass fiber-reinforced polymer composites was ~0.74 W/mK, which is nearly 3.5-fold lower than that of carbon-reinforced ones [33]. The use of ultrafine glass fibers of 0.5–3 µm in diameter [34] embedded in the rigid board offered excellent thermal insulating capabilities with a very low TC of 0.0287 W/mK. The major reason for this was its highly porous structure with a large volume of air in the glass fiber board. However, because glass fiber is an artificial pozzolan, similar to FCS, it is susceptible to PR in cement [35,36,37,38].

In cement, alkali hydrolysis from hydroxide ions attacking the fiber takes place, and the Ca(OH)_2_ reaction products precipitate on the fibers. This transforms tough and flexible fibers into brittle ones with poor durability. There exist alkali-resistant glass fibers [39,40]. In this study, we used hybrid glass/carbon fibers at a 67/33 mass ratio.

To minimize PR, cementitious materials with low pH would be required for FCS and E-glass fibers. In this study, calcium aluminum phosphate (CaP) cement, consisting of calcium aluminate cement (CAC) and sodium hexametaphosphate (SHMP, (NaPO_3_)_6_) as the chemically binding cement initiator, was adapted. CaP cement was modified with hybrid insulating aggregates and reinforcing micro-fibers. The pH of the pore solution extracted by centrifuging CaP cement slurries was around 10. Although this pH value is lower than the pH ~ 13 of conventional well cements, it may increase with increasing hydrothermal temperature. In fact, our previous work [41] on CaP cement containing Class F fly ash pozzolan demonstrated the formation of zeolite analcime (NaAlSi_2_O_6_·H_2_O), hydroxyapatite [HOAp, Ca_10_(PO_4_)_6_(OH)_2_], and boehmite (γ-AlOOH) at hydrothermal temperatures of 280 °C. Although HOAp was induced by acid–base reactions between the Ca^2+^ and OH^−^ liberated from hydrolysis of CAC and hydrolyzed SHMP, the formation of well-crystallized HOAp after extended hydrothermal exposure suggests continuous liberation of concentrated Ca^2+^ and OH^−^ ions, leading to a rise in pH. Correspondingly, the consumption of Ca^2+^ by chemical reactions with phosphate results in high concentrations of Na^+^ ions. As a result, analcime forms in the PR of aluminosilicate, such as mullite in fly ash, with Na^+^ and OH^−^. It is important to establish whether PRs of FCS shell and E-glass occur at temperatures lower than 280 °C. If so, the influence of such reactions and their products on mechanical behavior and thermal insulating properties of lightweight cement composites should be studied in samples made by autoclaving at 100, 175, and 250 °C.

Based upon the above information, the objective of this work was to evaluate the potential of hybrid HSA/FCS aggregates and glass/carbon fibers as thermal shock resistant and thermally insulating CaP cement composites in borehole energy storage and recovery systems at hydrothermal temperatures of 100, 175, and 250 °C.

The composite would need to have mechanical properties acceptable for geothermal wells, with a compressive strength of no less than 3.4 MPa.

One of the main challenges of formulating thermal insulating materials for underground reservoirs is their exposure to underground fluids, unlike insulating materials used in the construction industry. This study challenged the formulation and characterization of thermally insulating cementitious composites with a TC below that of water under water-saturated conditions.

## 2. Materials and Methods

### 2.1. Starting Materials

**Figure 1:**
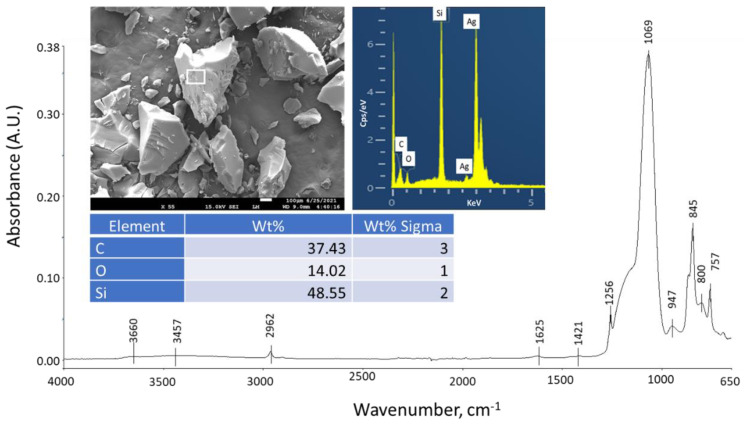
Appearance, elemental composition, and ATR-FTIR spectrum of HSA.

The details of the six starting materials are given in Table 1. In this experiment, we used: (1) Secar #71 calcium aluminate cement (CAC); (2) HSA as an ultralightweight aggregate with the trade name “Enova^®^ Aerogel IC3120 Particles” (Cabot Corp., Boston, MA, USA); (3) FCS as a lightweight aggregate with the trade name “CenoStar ES500” (CenoStar Corp., Newburyport, MA, USA); (4) Micro E-glass fiber (MGF, Fibertec Inc., Bridgewater, MA, USA) with the trade name “Microglass 7280”; (5) Micro carbon fiber (MCF, Asbury Graphite Mills, Inc., Asbury, NJ, USA) with the trade name “AGM-94”; (6) Sodium hexametaphosphate as a cement-forming reactant from Sigma-Aldrich (St. Louis, MO, USA). SHMP [(NaPO_3_)_6_], with a Calgon cyclic chain structure of six phosphate anions surrounded by six sodium cations, and a granular size of 75 µm. CAC was supplied by Imerys (Chesapeake, VA, USA); X-ray powder diffraction (XRD) data showed that the crystalline compounds of CAC included calcium monoaluminate (CaO·Al_2_O_3_, CA) and calcium dialuminate (CaO·2Al_2_O_3_, CA_2_). HSA, as amorphous silica, has particle sizes in the range of 0.1 to 1.2 mm, with a density of 0.12–0.15 g/cm^3^, and a thermal conductivity of 0.012 W/mK. Figure 1 presents a scanning electron microscope (SEM) image coupled with energy dispersive X-ray (EDX) elemental analysis and attenuated total reflectance Fourier transform infrared spectroscopy (ATR-FTIR) surveys for “as-received” HSA. Its elemental composition was 37.4% C, 14.0% O, and 48.6% Si (noting that Ag was used as a coating material to avoid charging of the sample surface). The detected C belongs to organic HMDS employed for the hydrophobic surface tailoring of the silica aerogel. HMDS-related agents undergo de-ammonia reactions, with the functional amine (=NH) group and silanol (≡Si-OH) group present on the silica surfaces forming trimethylsiloxy [-O-Si≡(CH_3_)_3_]-linked silica: 2≡SiOH + (CH_3_)_3_≡Si–NH–Si≡(CH_3_)_3_ (HMDS) → 2≡Si–O–Si≡(CH_3_)_3_ + NH_3_↑. This leads to the formation of a hydrophobic trimethylsilyl group [−Si≡(CH_3_)_3_] on the silica aerogel surfaces in conjunction with ammonia evolution [42,43,44].

The ATR-FTIR spectrum of HSA encompassed eleven absorption bands at 3660, 3457, 2962, 1625, 1421, 1256, 1069, 947, 845, 800, and 757 cm^−1^. The absorption bands at 3660 and 947 cm^−1^ [44,45,46,47] are assignable to stretching vibrations (*ν_O-H_*) and (*ν_Si-O_*) of O-H and Si-O bonds, respectively, reflecting that a certain amount of non-reacted silanol remains in HSA. The presence of hydrophilic silanol groups on HSA, treated with HMDS to make it hydrophobic, can help HSA disperse uniformly in aqueous cement slurry. The contributors to the 3457 and 1625 cm^−1^ absorption bands [47,48,49] are O-H asymmetric stretching (*ν_as O-H_*) and H-O-H bending (*δ_H-O-H_*) vibration modes, respectively, in water (H_2_O) molecules. The hydrophobic trimethylsilyl group-related bands [44,47,50,51] can be seen from methyl C-H asymmetric stretching (*ν_as C-H_*) and bending (*δ_C-H_*) modes in the Si–CH_3_ group at 2962 and 1421 cm^−1^, respectively, whereas the bands at 1256, 845, and 757 cm^−1^ belong to the Si-C bond in this group, namely, asymmetric stretching (*ν_as Si-C_*) at 1256 cm^−1^ and stretching (*ν_Si-C_*) for other bands. The band at 1256 cm^−1^ may overlap with silica gel. Two prominent absorption bands at 1069 and 845 cm^−1^ are from the Si-O-Si asymmetric and symmetric stretching, *ν_as_* *_Si-O-Si_* and *ν_s_* *_Si-O-Si_*, respectively, in trimethylsiloxy [-O-Si≡(CH_3_)_3_]-linked Si aerogel structure and Si aerogel itself.

**Figure 2:**
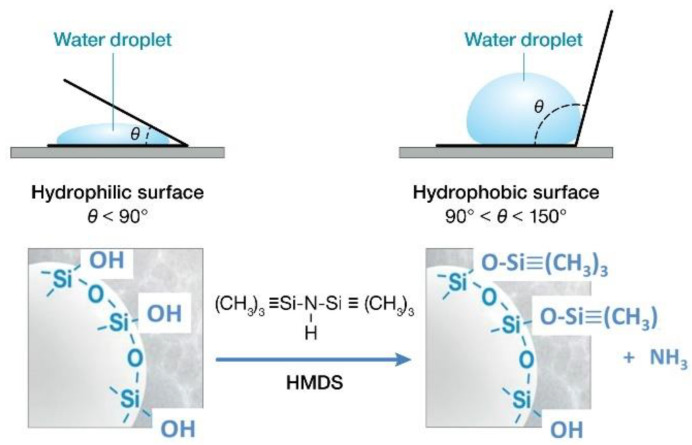
Hypothetical chemical structure and surface-wetting behaviors of non-treated and HDMS-treated HSA.

Based on these ATR-FTIR findings, a hypothetical schematic image for hydrophobically treated silica aerogel with HMDS can be drawn (Figure 2).

XRD analysis showed two crystalline phases in the composition of FCS, mullite (ICDD#04-016-1586, Al_2.22_Si_0.78_O_4.89_) and silica (#04-008-8437, SiO_2_). A 93% sodium metasilicate (SMS, Na_2_SiO_3_) powder with a particle size of 0.23 to 0.85 mm under the trade name “MetsoBeads 2048” was supplied by PQ Corporation (Avenel, NJ, USA) and had a 50.5/46.6 Na_2_O/SiO_2_ molecular weight ratio.

The FCS had a bulk density of 0.32–0.45 g/cm^3^ and λ of 0.1–0.2 W/mK. The cumulative size distribution of FCS was as follows: 3 wt.% 300 µm, 54 wt.% 150 µm, 19.5 wt.% 106 µm, 15 wt.% 75 µm, and 8.5 wt.% < 74 µm.

To improve compressive toughness, which suppresses crack development and propagation, non-crystalline MGF were used. The fibers were 16 µm in diameter and 120 µm in length and had a bulk density of 0.93 ± 0.08 g/cc. MCF derived from a polyacrylonitrile (PAN) precursor was 7–9 µm in diameter and 100–200 µm in length. The visual appearance of both MGF and MCF was powder-like products.

### 2.2. Cement Formulas and Samples Preparation

In our study, the FCS/HSA aggregate wt. ratios were 100/0, 94/6, 90/10, 88/12, and 83/17, whereas the CAC/total aggregate wt. ratio was 70/30 for all formulations. The content of MGF and MCF was 10 and 5 wt.%, respectively, by total weight of CAC, FCS, and HSA. SHMP was used at 6 wt.% by total weight of CAC in all aggregates. All solid ingredients described above were prepared as a dry blend prior to mixing with water.

The composite and reference samples were prepared in the following sequences. A certain amount of water was added to a dry blended composite, followed by hand mixing for 1 min. The hand-mixed slurry was poured into molds (the mold shape depended on the test) and was left for 24 h at ambient temperature, allowing it to initiate acid–base reactions. Thereafter, the hardened composite was removed from the molds and placed in a 99 ± 1% relative humidity environment for 24 h at 85 °C. Finally, the pre-cured composite was autoclaved in a non-stirred Parr Reactor 4622 (Hillsboro, OR, USA) for 24 h at 100, 175, and 250 °C.

### 2.3. Measurements

Assuming that CaP cement is used in storage wells at the medium and high temperatures of 100 and 250 °C, the thermal shock (TS) test was carried out by heating the 100- or 250 °C-autoclaved CaP composite samples for 24 h in an oven at 175- or 250 °C, respectively, followed by immersion of hot cement in 25 °C water. This heat-quenching process was repeated three times. Therefore, in the 175 and 250 °C storage environment, the thermal shock-related temperature gradients were set at 150 and 225 °C, respectively. TS resistance was evaluated based on the changes in compressive strength and TC after the TS.

To evaluate the hydrothermal stability of HSA, we autoclaved HSA for 24 h at 100, 175, and 250 °C, and visually checked its miscibility with water. We also determined the water droplet contact angle on autoclaved HSA samples after 100 °C drying and conducted ATR-FTIR analyses. Water droplet contact angle measurements at ambient temperatures were assessed using Model CAA 3, Imass Inc. (Marshfield, MA, USA).

Pellet samples, 13.0 mm diameter by 1.7 mm height, were prepared for contact angle measurements according to the following sequence: (1) An amount of 0.6 mg HSA was blended with 15.0 g deionized water in a 20 mL sealed glass ampoule at ambient temperature; (2) The ampoule was autoclaved for 24 h at 100, 175, and 250 °C; (3) The autoclaved HSA was dried for 24 h in an oven at 100 °C; (4) An amount of 0.2 mg of dried sample was placed in the CrushIR™ 15 Ton Hydraulic Press (Fitchburg, WI, USA) mold; (5) A 10 ton pressure was loaded over the powder to make pellet-shaped samples. The dried autoclaved samples were also used in ATR-FTIR and TGA/DTA analyses. The former analysis was aimed at investigating the hydrothermal stability of the hydrophobic trimethylsiloxy group linked to silica aerogel. The latter was used to collect the thermal decomposition parameters, including the overall decomposition pattern, onset decomposition temperature, and weight losses. TGA/DTA (model Q50, TA Instruments, New Castle, DE, USA) analyses were run at a heating rate of 10 °C/min in a N_2_ flow.

The slump flow of the cement slurries was measured by using a polyethylene flow cone with a top hole with a diameter of 20 mm, bottom hole with a diameter of 45 mm, and a height of 40 mm. The cement slurry was filled in the cone and placed on a flat carbon steel plate. Thereafter, the cone was slowly lifted, allowing the slurry to flow freely. The slurry slump, in mm, was determined 20 s after flow onset.

For compressive strength and compressive toughness, the composite samples were prepared in cylindrical molds (20 mm in diameter and 40 mm in height). Electromechanical Instron System Model 5967 (Norwood, MA, USA) was used to obtain these mechanical properties. Compressive strength is the capacity of a material or structure to withstand under compression. Cement with high compressive strength may be brittle and lack stress energy absorption, resulting in the rapid propagation of pre-existing and newly created cracks in cement bodies under stress conditions. Thus, cement ideally possesses toughness that delays or prevents crack propagation. To obtain quantitative data on compressive toughness, we determined the total energy consumed during the completion of cement compressive failure; this was computed from the enclosed area of the compressive stress–strain curve with the baseline extending from the beginning to the end of the curve. Toughness depended primarily on the balance between compressive strength and ductility.

To investigate the pozzolanic activity of each insulating aggregate and micro-fiber reinforcement, the 100, 175, and 250 °C-autoclaved CaP cements, containing one of FCS, HSA, MGF, and MCF, were prepared and studied using XRD, ATR-FTIR, and SEM-EDX probes. Changes in mechanical behavior due to pozzolanic activity were assessed by compressive-strength and compressive-toughness measurements.

To obtain information on the hydration of CaP composite slurries with different FCS/HSA ratios, a calorimetric study was done at the isothermal temperatures of 25, 50, and 85 °C, using TAM air isothermal microcalorimetry (TA Instruments, New Castle, DE, USA). We determined the initial and final setting times and heat energy evolved during the acid–base and hydration reactions.

TC was measured with the Quick Thermal Conductivity Meter, QTM-500, Kyoto Electronic (Kyoto, Japan) on rectangular prism samples (60 mm wide, 120 mm long, and 20 mm thick). Measuring TC in hydrous conductive material such as cement requires the use of a water-proofed probe consisting of a single heater and thermocouple. We placed the probe on the cement surface after removing the excess water on the water-saturated cement using a dry paper towel.

XRD (40 kV, 40 mA copper anode X-ray tube) and ATR-FTIR were used to identify amorphous and crystalline phase compositions and phase transitions of tested samples. The PDF-4/Minerals 2021 database of the International Center for Diffraction Data (ICDD) was used to analyze the XRD patterns.

JEOL 7600F SEM (Pleasanton, CA, USA) image analysis coupled with EDX elemental composition survey were used in this work.

The experiment included seven steps: (1) Assessment of hydrothermal stability of HSA at temperatures of up to 250 °C; (2) Investigation of workability and pumpability of the CaP composite slurry by slump and slurry density measurements; (3) Understanding pozzolanic and mechanical behaviors of individual insulating aggregates and micro-fibrous reinforcements in CaP composite system after autoclaving at 100, 175, and 250 °C; (4) Identification of cement-forming pathways by following exothermic chemical and hydration reactions of CaP composite slurries as a function of the FCS/HSA ratio at 85 °C; (5) Evaluation of compressive-strength and compressive-toughness of 100 and 250 °C-autoclaved CaP cement composites, before and after thermal shock (TS) tests; (6) Determination of TC and water-saturated bulk density as a function of the FCS/HSA ratio for 100 and 250 °C-autoclaved pre- and post-TS samples; (7) Study of phase composition and transitions along with microstructures and their alterations for an optimized composite formula.

## 3. Results and Discussion

### 3.1. Hydrothermal Stability of HSA

**Figure 3:**
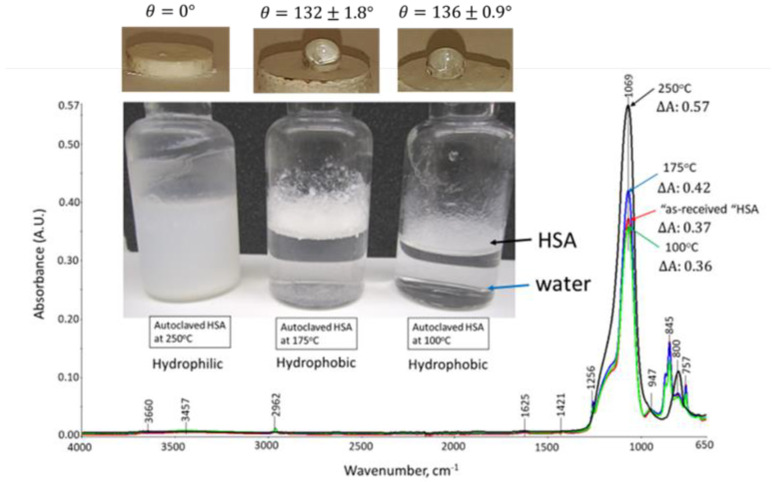
Visual characteristics, water repellency, and ATR-FTIR analyses for 100, 175, and 250 °C-autoclaved HSA.

Figure 3 shows the phase separation for HSA and water, water droplets on the HSA surfaces, and ATR-FTIR analysis of HSA samples autoclaved at 100, 175, and 250 °C. Both 100 and 175 °C samples show water–HSA phase separation and water repulsion by the hydrophobic surfaces of HSA exposed to these temperatures for 24 h. In contrast, at 250 °C, water and HSA form a uniform mixture and water spreads over the HSA surface, suggesting that HSA has become hydrophilic at this elevated temperature. In fact, the contact angle, *θ*, of the water droplet on compressed dry HSA surfaces after the autoclaving for 24 h was 135 ± 1.5 (not shown), 136 ± 0.9, and 132 ± 1.8 degrees (°) for “as received” HSA, 100, and 175 °C-autoclaved samples, respectively. The water repellent behavior of solid surfaces based on the contact angle, in degrees (°), is categorized as follows [52]: superhydrophobic > 150°, over-hydrophobic between 150 and 120°, hydrophobic 120–90°, and hydrophilic < 90°. Thus, the surfaces of all samples except for the 250 °C-autoclaved sample could be categorized as over-hydrophobic.

As expected, it was very difficult to measure the contact angle for 250 °C-autoclaved HSA; in fact, the compressed sample allowed the water to permeate through it, thereby no longer showing hydrophobicity. ATR-FTIR results supported this observation of transformation from a hydrophobic to hydrophilic sample. Namely, all hydrocarbon (CH_3_)-related bands at 2962, 1421, 1256, 845, and 757 cm^−1^ in the hydrophobic trimethylsilyl group vanished after 250 °C-autoclaving. Furthermore, hydrophilic silanol-related bands at 3457 and 947 cm^−1^ were also eliminated, whereas the peak height (∆A) of two Si-O-Si linkage-related bands at 1069 and 800 cm^−1^ noticeably rose, compared with that of “as-received”, 100, and 175 °C samples. For instance, ∆A at 1069 cm^−1^ for 250 °C-treated HSA was 0.57, corresponding to 54, 62, and 36% higher values than “as-received”, 100, and 175 °C-autoclaved samples, respectively. Although there is no experimental evidence, it is possible to rationalize that at 250 °C, the scission of -Si≡(CH_3_)_3_ bonds in the trimethylsiloxy-linked silica aerogel structure takes place: ≡Si-O-Si≡(CH_3_)_3_ → ≡Si-O-Si//(CH_3_)_3_ [53]. The cleavage site may be susceptible to hydroxylation with the formation of new silanols: ≡Si-O-Si//(CH_3_)_3_ + 3H_2_O → ≡Si-O-Si≡(OH)_3_ + 3CH_4_ (gas)↑ [54]. Next, self-condensation between silanols, ≡Si-O-Si≡(OH)_3_ + (HO)_3_≡Si-O-Si≡ → ≡Si–O–Si≡(O)_3_≡Si-O-Si≡  + 3H_2_O, may occur to form silicon-like three-dimensional polymers with additional Si-O-Si linkages [55,56,57,58]. If so, such self-condensation explains why the peak height of Si-O-Si bands remarkably increased, whereas silanol bands disappeared.

**Figure 4:**
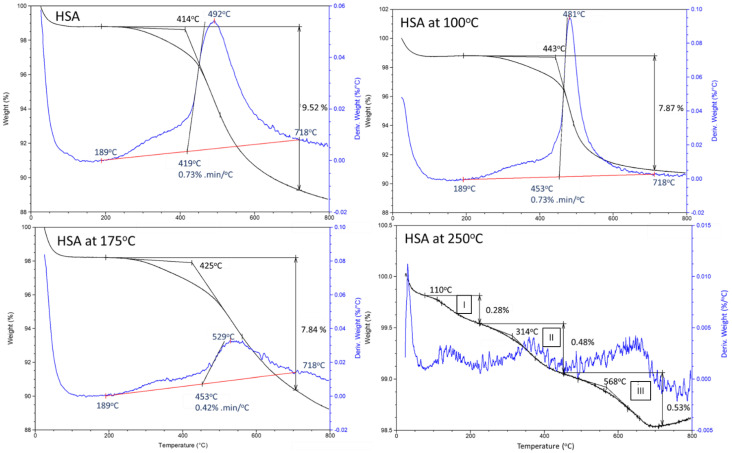
TGA/DTG analyses for 100, 175, and 250 °C-autoclaved HSA.

Additionally, a TGA survey in the temperature range of 25–800 °C supported the above information. Figure 4 shows TGA and DTG curves of “as-received” HSA as control, and 100° 175, and 250 °C-autoclaved HSA samples. All the TGA curves except for the 250 °C sample showed a one stage-decomposition pattern with a degradation onset point at ~414 °C. The decomposition onset and end points were determined from the DTG curves. The total mass loss rate (TMLR, %/mm) was computed from the area under the TGA curve, between the onset and end points. The weight loss (WL, %) was estimated from the TGA curve. For “as-received” HSA, it is possible to assume that the WL of ~9.52% was due to the decomposition of all trimethylsilyl groups. Similar curves, except for the smaller WL of ~7.87%, was observed for 100 °C-autoclaved sample, suggesting that ~1.65% of hydrocarbons may be decomposed during autoclaving at this temperature. At 175 °C, even though the WL value was almost the same as that at 100 °C, the TMLR declined from 0.73 (at 100 °C) to 0.42%/min. It is likely that the carbonaceous degradation products of CH_3_ in the trimethylsilyl groups contributed to the WL. This suggests that some trimethylsilyl groups might be degraded at 175 °C, but HSA remains hydrophobic. The sample autoclaved at 250 °C displayed a three-stage decomposition (I, II, III in the Figure 4) with onset points at 110, 314, and 568 °C. The WL at each stage was 0.28, 0.48, and 0.53%. As aforementioned in the ATR-FTIR study, the hydrophobic trimethylsilyl groups at 250 °C underwent three steps of molecular alterations: (1) Scission of the -Si≡(CH_3_) bond; (2) Substitution of the -CH_3_ group for a hydroxyl (-OH) group, to form silanol -Si≡(OH)_3_; (3) Self-condensation between silanols, making it possible to assemble a silicon-like polymer. Therefore, these three degradation stages may involve the degradation of silicon-like polymer resulting in a total WL of 1.29%.

### 3.2. Slurry Properties

Table 2 gives the properties of CaP slurries as a function of the FCS/HSA ratio, including the water/blend (W/B) weight ratio (blend weight includes that of CAC, FCS, and HSA), density, slump size, and pH of pore solution extracted from the slurry by centrifuging. The W/B ratio was decided based on the addition of water up to the point where some water was pushed to the surface of the slurry, a phenomenon called “water bleeding”. As the water uptake of the reference slurry without HSA was the same as the slurry with the highest HSA content, it can be concluded that HSA did not take up any additional water, due to the hydrophobic treatment of its surface. The cement slurries were made with W/B ratios in the range of 0.48–0.55. As expected, a downward trend for slurry density was observed; the density declined with increasing HSA content, from 1.29 g/cm^3^ at FCS/HSA 100/0 to 1.07 g/cm^3^ at 83/17. Similar to the W/B ratio, no specific trend was observed for slump size; it was in the range between 78 and 83 mm for all samples. Such slurry properties indicate that all the aggregates and reinforcements were uniformly distributed in a slurry of good fluidity, which is important for the pumpability of the blend in the field.

### 3.3. Pozzolanic and Mechanical Behaviors of Aggregates and Reinforcements

To understand pozzolanic and mechanical behaviors of individual insulating aggregates and micro-fibrous reinforcements in the CaP composite system, after autoclaving at 100, 175, and 250 °C, four CaP cement composites, each with one of FCS, HSA, MGF, and MCF, were prepared. These blends had 24.9 g FCS, 5.1 g HSA, 10 g MGF, and 5 g MCF, each mixed with 70 g CAC containing 6 g SHMP. For comparison purposes, neat CaP cement without any aggregates or reinforcements was prepared as a control.

#### 3.3.1. Neat CaP Cement

**Figure 5:**
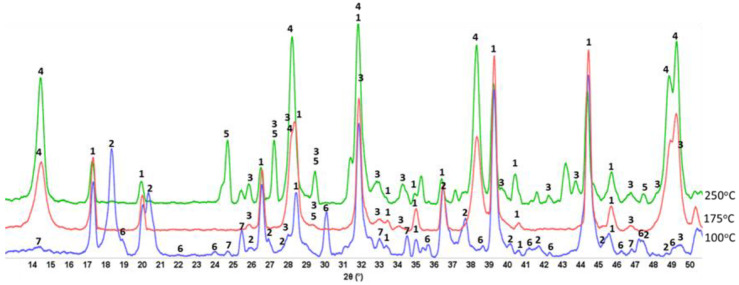
Diffractograms for 100-, 175-, and 250 °C-autoclaved neat CaP cements: 1-katoite (ICDD#00-024-0217/01-077-0240/01-084-01354/01-074-9779); 2-gibbsite (01-076-1782); 3-hydroxyapatite (04-016-1647/04-011-6221/01-074-9779); 4-boehmite (04-010-5683); 5-calcium carbonate (01-085-6719); 6-krotite (00-023-1037); and 7-grossite (00-023-1037).

Figure 5 presents XRD patterns of neat CaP cement (pore solution of pH 9.77) after autoclaving for 24 h at 100, 175, and 250 °C. Three major crystalline phases were identified in the patterns of 100 °C-autoclaved samples: Si-free katoite (C_3_AH_6_), gibbsite (AH_3_), and monocalcium aluminate (CA), krotite. Additionally, two minor phases were calcium dealuminate (CA_2_), grossite, and hydroxyapatite [(HOAp, Ca_10_(PO_4_)_6_(OH)_2_]. Hydration of CA and CA_2_ present in CAC resulted in the formation of Si-free katoite and gibbsite through the following reactions: 3CA + 12H_2_O → C_3_AH_6_ + 2AH_3_ and 3CA_2_ + 21H_2_O → C_3_AH_6_ + 5AH_3_ [59,60]. Thus, some non-hydrated clinkers remained in the 100 °C-24 h-autoclaved neat CaP cement. It appears that cement slurry made from the dry blend of CAC and SHMP at 100 °C provides two different cementitious binding phases: (1) CAC hydration products, such as katoite and gibbsite; (2) HOAp, the reaction product of SHMP with CAC. At 175 °C, the features of the XRD patten were strikingly different from those at 100 °C. There were four major differences: (1) Non-hydrated CA and CA_2_ clinkers at this temperature were not found, suggesting that more alkalis such as Ca^2+^ and OH^−^ were dissociated from CA and CA_2_; (2) Disappearance of all gibbsite-related *d-spacings*, whereas the line intensity of katoite remained unchanged; (3) Emergence of boehmite (γ-AlOOH) phase high-intensity peaks; (4) Increased intensity of HOAp-related peaks. Increased content calcium and hydroxide ions from CAC hydration resulted in higher HOAp concentrations and higher intensities of its peaks. As for (2) and (3), the alkali dissolution and recrystallization of gibbsite produces boehmite, Al(OH)_3_ + OH^−^ → Al(OH)_4_^−^ → AlH(OH)_4_ → γ-AlOOH + 2H_2_O [61]. Hence, three major crystalline phases, HOAp, katoite, and boehmite, were responsible for strengthening neat CaP cement at 175 °C. A similar pattern, except for the appearance of intense line signals of calcium carbonate (CaCO_3_), was observed for the 250 °C-autoclaved sample.

**Figure 6:**
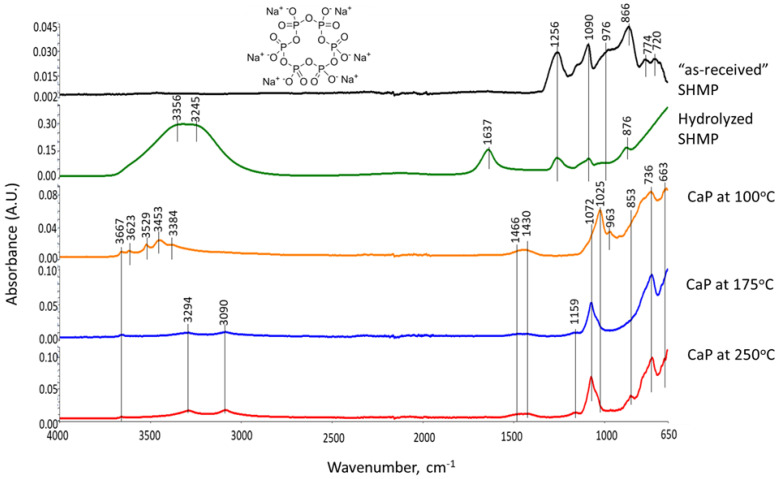
ATR-FTIR absorption spectra for “as-received” SHMP salt, hydrolyzed SHMP solution, and 100-, 175- and 250 °C-autoclaved neat CaP cements.

These XRD results were supported by the ATR-FTIR results. Figure 6 depicts ATR-FTIR spectra in the frequency region of 4000 to 650 cm^−1^ for “as-received” Calgon SHMP salt, hydrolyzed SHMP solution, and 100-, 175-, and 250 °C-autoclaved neat CaP cements. For “as-received” SHMP, the two bands located at 774 and 720 cm^−1^ represent the P-O-P linkage in the Calgon cyclic chain structure [62]. The other bands at 1256, 1090, 976, and 866 cm^−1^, respectively, are due to asymmetric stretching (*ν_as P=O_*) of the P=O bond, asymmetric (*ν_as P-O_*) and symmetric (*ν_s P-O_*) vibration modes of the P-O bond, as well as symmetric (*ν_s P-O-P_*) of the P-O-P linkage in Calgon [62,63,64,65]. Compared with these results, the spectral features of hydrolyzed SHMP exhibited three differences: (1) Disappearance of all cyclic P-O-P linkage-associated bands at 774 and 720 cm^−1^; (2) Shift of the 866 cm^−1^ band of *ν_s P-O-P_* towards a higher frequency site at 876 cm^−1^; (3) Presence of water-related bands at 3356, 3245, and 1637 cm^−1^, whereas no shift was observed from *ν_as P=O_*, *ν_as P-O_*, and *ν_s P-O_* bands. For the first two differences, it is possible to rationalize that the hydrolysis of SHMP led to cycle-opening and cycle-scission, thereby initially transforming the Calgon into ionic liner polyphosphate, which was further hydrolyzed to orthophosphate [62,66,67,68,69,70]. The pH of SHMP solution was ~6.5. As the predominant orthophosphate species at pH~6.5 are H_2_PO_4_^−^ and HPO_4_^2−^ [70], these anions likely play a major role in promoting acid–base reactions with Ca^2+^ alkali hydrolysate from CAC.

As described in the XRD study, the 100 °C-autoclaved neat CaP cement had two major crystalline phases: Si-free katoite and gibbsite, derived from the hydration of CAC. Correspondingly, the ATR-FTIR spectrum indicated the presence of these hydration products. The spectrum involved three groups: (1) OH in the ranges of 3700–3300 cm^−1^ and 1100–900 cm^−1^; (2) Condensed tetrahedral AlO_4_ in 800–700 cm^−1^; (3) Condensed octahedral AlO_6_ in 680–650 cm^−1^. Regarding gibbsite [71,72], the O-H bond stretching (*ν_O-H_*) and O-H bending (*δ_O-H_*) vibration modes involved six frequencies, with 3623, 3529, 3453, and 3384 cm^−1^ belonging to *ν_O-H_*, and 1025 and 963 cm^−1^ as *δ_O-H_*, whereas the Al-O bond stretching (*ν_Al-O_*) in tetrahedral AlO_4_ and octahedral AlO_6_ groups can be recognized by the presence of 736 and 663 cm^−1^ bands, respectively. The bands at 1466, 1430, and 853 cm^−1^ are related to CO_3_^2−^ group in carbonate compounds; namely, the first two bands are assignable to *ν_as C-O_* in CO_3_^2−^ and the last one is O-C-O out-of-plane bending (*δ_O-C-O_*) [73,74]. As crystalline carbonate was not found by XRD, the detected carbonate is likely amorphous. Additionally, the presence of HOAp as a minor phase in XRD can be identified from a shoulder band at 1072 cm^−1^ attributed to the *ν_as P-O_* of the PO_4_^3−^ group [62,75,76].

On the other hand, Si-free katoite-related bands [77,78,79] involved the stretching (*ν_O-H_*) mode at 3667 cm^−1^. The contributors to the other four bands at 1025, 963, 736, and 663 cm^−1^ were the same as those of gibbsite. At 175 °C, there were three major changes in the spectrum compared with 100 °C. First, there was a disappearance of all gibbsite-related bands, whereas Si-free katoite-related bands remained unchanged. Secondly, there was an emergence of three new principal bands at 3294, 3090, and 1159 cm^−1^. Thirdly, the shoulder band at became the principal one. The three new bands were associated with the *ν_as O-H_*, *ν_s O-H_*, and *δ_O-H_* of the OH group in boehmite [80,81,82]. The shoulder band at 1072 cm^−1^ was relevant to the *ν_as P-O_* of the PO_4_^3−^ group in HOAp, as the major phase. All the results agreed with the XRD findings. Spectral features of the 250 °C-autoclaved sample closely resembled the spectrum at 175 °C.

**Figure 7:**
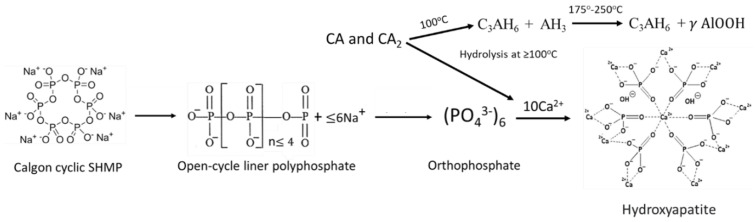
Illustration of cement-forming acid–base and hydration reactions in neat CaP cement.

Thus, the three major phases, HOAp, boehmite, and Si-free katoite, were formed in neat CaP cement in the range from 175 to 250 °C. At 100 °C, HOAp, as a minor phase, was induced by acid–base reactions between the orthophosphoric acid liberated from hydrolyzed SHMP and Ca^2+^ and OH^−^ alkali hydrolysates of CA and CA_2_. Higher temperatures promoted more acid formation from SHMP and alkalis from CA and CA_2_, resulting in the HOAp becoming the major phase. Aluminum from CA and CA_2_ formed gibbsite at 100 °C, which transferred to boehmite at 175 °C. Based on the above information, Figure 7 illustrates two different cement-forming pathways by acid–base and hydration reactions in neat CaP cement.

**Figure 8:**
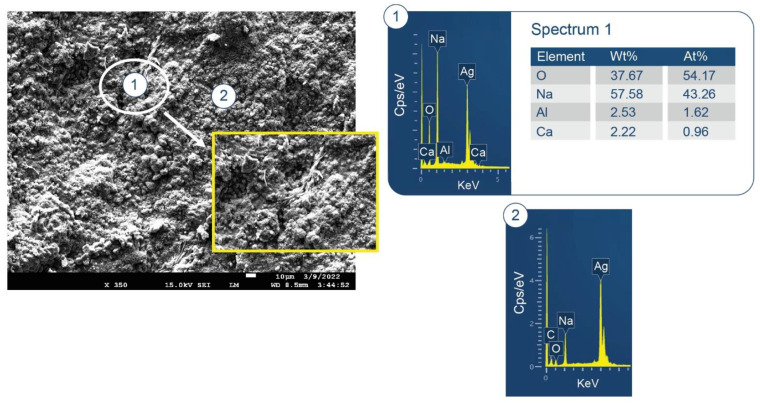
Morphology and elemental composition of freshly fractured neat CaP cement after curing at 100 °C.

As seen in Figure 7, the formation of HOAp as the major phase engenders release of Na^+^ ions due to the consumption of orthophosphate. Simultaneously, hydrolysis of CA and CA_2_ creates an alkaline environment through the release of Ca^2+^ and OH^−^. This is the reason why the reference pH of neat CaP is 9.77. The high pH raises concerns about activating pozzolanic decomposition of lightweight aggregates and glass fibers. In fact, as seen in SEM-EDX data (Figure 8) on a freshly fractured surface of 100 °C-autoclaved CaP, the predominant element in areas marked as No. 1 and 2 is Na, representing more than half of the total mass weight (wt%) of all elements.

Pozzolanic reactions of FCS, HSA, and MGF in alkaline environments of CaP slurries after curing at 100, 175, and 250 °C were investigated in the next step. A formulation with a non-pozzolan MCF reinforcement was used as a control.

#### 3.3.2. CaP/FCS System

**Figure 9:**
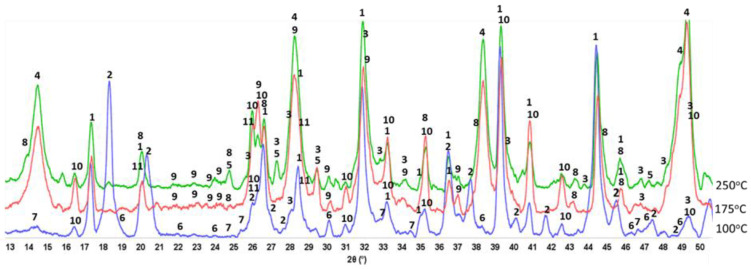
XRD patterns for 100-, 175-, and 250 °C-autoclaved CaP/FCS cements: 1-katoite (00-024-0217/01-077-0240/01-084-01354/01-074-9779); 2-gibbsite (01-076-1782); 3-hydroxyapatite (04-016-1647/04-011-6221/01-074-9779); 4-boehmite (04-010-5683); 5-calcium carbonate (01-085-6719); 6-krotite (00-023-1037); 7-grossite (00-023-1037); 8-hydrosodalite (00-040-0102); 9-albite (01-089-6427); 10-mullite (04-016-1586); and 11-silicon oxide (00-014-0654).

Figure 9 shows XRD patterns for 100-, −175-, and −250 °C-autoclave CaP/FCS cements (pore solution pH 10.06). The features of 100 °C-autoclaved CaP/FCS and neat CaP cement were alike, except for the two FCS-associated crystalline phases, mullite (ICDD#04-016-1586) and silica (#00-014-0654). At 175 °C, similar to neat CaP, the two non-hydrated phases, CA and CA_2_, disappeared and HOAp became the major phase. The gibbsite phase was transferred to the boehmite phase, whereas katoite remained a major phase. The only difference from the neat cement was the appearance of low-intensity albite (NaAlSi_3_O_8_) peaks in the presence of FCS. The reaction of albite formation is Na^+^ + Al(OH)_4_^−^ +3H_4_SiO_4 (*aq*)_ ↔ NaAlSi_3_O_8_ + 8H_2_O [83]. Orthosilicic acid (H_4_SiO_4_) was possibly derived from the alkaline dissolution of silica and aluminosilicate mullite in the FCS shell, whereas Al(OH)_4_^−^ came from the hydrolysis of CAC. Thus, FCS appears to undergo PR; namely, alkali dissolution of the FCS shell leading to the precipitation of albite, a PR product. At 250 °C, another PR product was identified in addition to albite: zeolite hydrosodalite [Na_6_(AlSiO_4_)_6_·8H_2_O]. However, strong peaks of mullite and silica were still present in the 250 °C XRD pattern, suggesting that a certain amount of these pozzolans remained unreacted.

**Figure 10:**
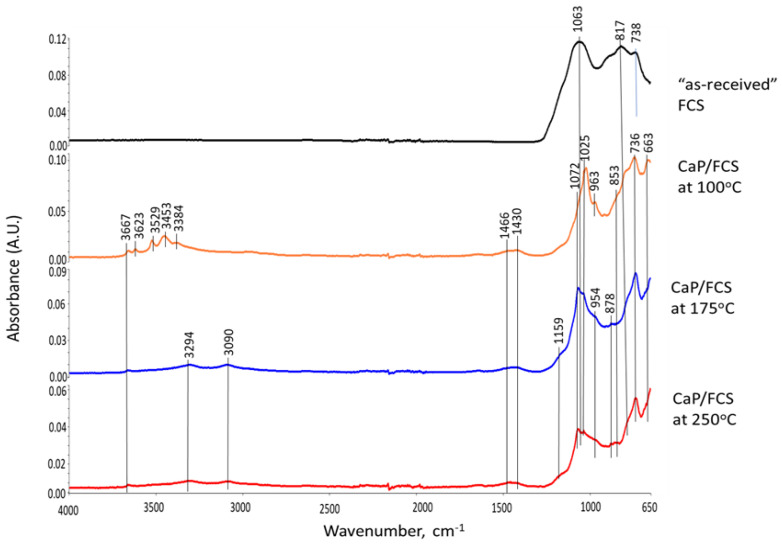
ATR-FTIR spectra for 100-, 175-, and 250 °C-autoclaved CaP/FCS cements.

Figure 10 gives ATR-FTIR spectra for 100-, 175-, and 250 °C-autoclaved CaP/FCS cements. In agreement with the XRD results, the principal crystalline phases of FCS, mullite and silica, are represented by the bands at 1063 and 817 cm^−1^, corresponding to the M-O (M: Si or Al) anti-symmetric (*ν_as M-O_*) and symmetric (*ν_s M-O_*) stretching vibrations, respectively, in the Si-O-Al network of mullite and Si-O-Si linkage of silica [84,85]. The 745 cm^−1^ band is the Si-O stretching (*ν_s Si-O_*) mode in crystalline and amorphous silica [86,87]. The spectra of CaP/FCS and neat CaP blends after 100 °C-autoclaving were similar. At 175 and 250 °C, the same major reaction products—HOAp, boehmite, and katoite—were in both CaP/FCS and neat CaP systems. The difference of spectral patterns was in the appearance of two new weak bands at 954 and 878 cm^−1^. In our previous study on the interactions between fly ash F and sodium metasilicate at 175 °C [1], the M-O (M: Si and Al) (*ν_as M-O_*) in two crystalline Na_2_O-Al_2_O_3_-SiO_2_-H_2_O (N-A-S-H) systems, Na(Zeolite P) and analcime, were identified at 1005 and 870 cm^−1^ bands, respectively, in conjunction with XRD findings. These new bands in the region of 1000 to 870 cm^−1^ can be attributed not only to the formation of crystalline N-A-S-H compounds induced by the PR of mullite and silica in FCS with Na^+^ and OH^−^ alkalis, but also to the amorphous and crystalline CaO-Al_2_O_3_-SiO_2_-H_2_O (C-A-S-H) and CaO,Na_2_O-Al_2_O_3_-SiO_2_-H_2_O (C,N-A-S-H) hydrates and anhydrates assembled by the PR of FCS with Ca^2+^ and OH^−^ ions released by hydrolysis of CAC [88,89,90].

In summary, in agreement with the XRD results, FCS was susceptible to PR at hydrothermal temperatures of ≥175 °C. As a result, the albite (anhydrous N-A-S system) and hydrosodalite zeolite (N-A-S-H system) were present as minor phases in the cement matrix along with the major phases of HOAp, boehmite, and katoite. Thus, the 663 cm^−1^ band belonged not only to katoite, but also overlapped with the M-O (M: Si and Al) (*ν_as M-O_*) in albite and hydrosodalite.

**Figure 11:**
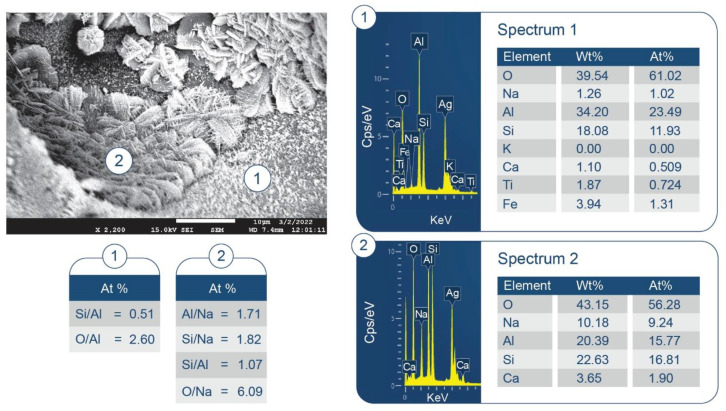
Morphology and elemental composition of freshly fractured neat CaP/FCS cement after curing at 250 °C.

**Figure 12:**
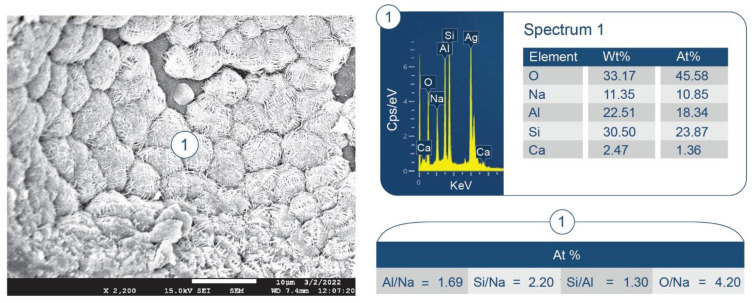
Morphology and elemental composition of pozzolanic reaction products on the surface of FCS of CaP/FCS cement cured at 250 °C.

**Figure 13:**
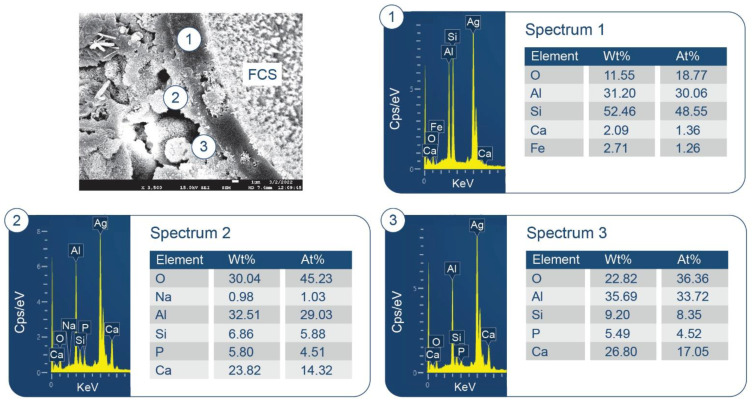
Morphology and elemental composition at the interface between cement and FCS surface of CaP/FCS cement cured at 250 °C.

SEM/EDX analysis of the 250 °C-autoclaved sample is shown in Figure 11. At location No. 1, Si/Al and O/Al atomic ratios were 0.51 and 2.60, respectively. The FCS shell includes mullite (Al_2.22_Si_0.78_O_4.89_) and silica (SiO_2_). The Si/Al and O/Al atomic ratios of mullite were 0.35 and 2.20, respectively. Thus, these spiky crystals were identified as mullite in FCS. The ~10 µm porous cotton ball-like crystals formed in the vicinity of FCS involved Na as one of its major elements. Their atomic ratios of Al/Na, Si/Na, Al/Si, and O/Na were 1.71, 1.82, 1.07, and 6.09, respectively. Among PR products, the hydrosodalite [Na_6_(AlSiO_4_)_6_8H_2_O] had similar atomic ratios of Al/Na = 1.0, Si/Na = 1.0, Al/Si = 1.0, and O/Na = 5.3 (Na is commonly underestimated in EDX analyses). Likewise, copious amounts of hydrosodalite crystals (less than ~5 µm in size) with similar morphology formed over FCS surfaces (Figure 12). Figure 13 shows an image of cement adhering to the FCS surface. The composition of the rim-like structure of ~4 µm thickness includes three major elements, Al, Si, and O, typical of a FCS shell. Cement is well-adhering to the FCS shell (locations No. 2 and 3). In location No. 2, the elemental composition of cement includes O, Al, and Ca as major elements and P and Si as minor ones. The Ca/Al, Si/Al, and Ca/Al atomic ratios of amorphous C-A-H and C-A-S-H systems in this location were 0.49, 0.20, and 2.43. The P is related to HOAp. Similar atomic ratios were observed in location No. 3: Ca/Al = 0.51, Si/Al = 0.25, and Ca/Si = 2.04. The presence of Ca in the reaction products covering FCS strongly suggests that the FCS surface was susceptible to PR, not only with Na^+^, but also with Ca^2+^ ions. Relating this to ATR-FTIR results, it appears that amorphous C-A-H and C-A-S-H also contribute to the bands in the region of 1000 to 870 cm^−1^.

Some typical hydrosodalite-related porous crystalline balls were seen in the cement/FCS interfacial boundary region and at locations adjacent to FCS. Hence, the pozzolanic activity of FCS offered improved interfacial bonding of FCS with cement through the formation of crystalline albite, hydrosodalite, and amorphous C-A-H and C-A-S-H PR products.

#### 3.3.3. CaP/HSA System

**Figure 14:**
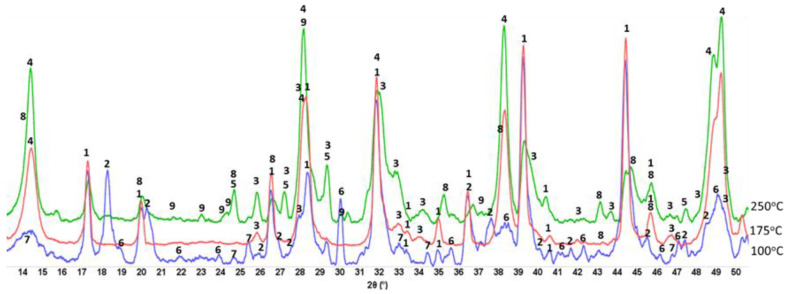
XRD patterns for 100-, 175-, and 250 °C-autoclaved CaP/HSA cements: 1-katoite (00-024-0217/01-077-0240/01-084-01354/01-074-9779); 2-gibbsite (01-076-1782); 3-hydroxyapatite (04-016-1647/04-011-6221/01-074-9779); 4-boehmite (04-010-5683); 5-calcium carbonate (01-085-6719); 6-krotite (00-023-1037); 7-grossite (00-023-1037); 8-hydrosodalite (00-040-0102); and 9-albite (01-089-6427).

Figure 14 shows the XRD patterns of 100-, 175-, and 250 °C-autoclaved CaP/HSA cements (pore solution pH 10.74). At 100 and 175 °C, the major crystalline products of this system were very similar to those in neat CaP. At 100 °C, they included katoite and gibbsite, and at 175 °C, HOAp, boehmite, and katoite. Unlike the CaP/FCS system, N-A-S type minerals, albite, and N-A-S-H phases were not identified at 175 °C. The TGS/DTG results suggested some degradation of trimethylsilyl groups in HSA at 175 °C. If the loss of trimethylsilyl groups was significant, the PR of HSA with sodium could proceed with the formation of N-A-S or N-A-S-H type reaction products. As such products were not detected, it is reasonable to assume that even with some degradation of trimethylsilyl groups, the PR of HSA were negligible at 175 °C. In contrast, because all hydrophobic trimethylsilyl groups were decomposed at 250 °C, the trimethylsilyl-free hydrophilic silica aerogels became susceptible to alkali dissolution, similar to FCS. The XRD patterns of the 250 °C-autoclaved sample included peaks of albite and hydrosodalite brought about by the hydrothermal interactions of the following three reactants: (1) Na^+^; (2) Al(OH)_4_^−^ from the hydrolysis of CAC; (3) (H_4_SiO_4_)_(*aq*)_, derived from the alkali dissolution of silica gel.

**Figure 15:**
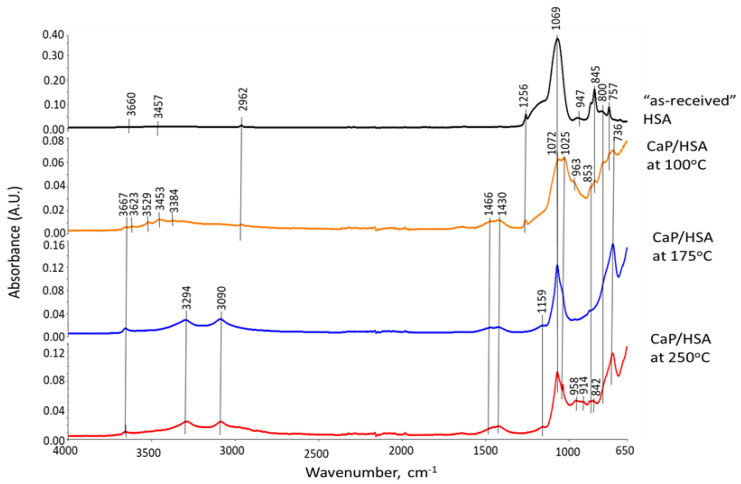
ATR-FTIR spectrum analysis of 100-, 175-, and 250 °C-autoclaved CaP/HSA system.

ATR-FTIR results (Figure 15) at 100 °C detected all HSA-related bands coexisting with gibbsite and katoite as major phases, clearly demonstrating that HSA was not susceptible to PR at that temperature. However, at 175 °C, all CH_3_-related bands in trimethylsilyl disappeared. As mentioned in previous hydrothermal stability studies of HSA, HSA exposed to 175 °C in deionized water retained CH_3_ bands. However, trimethylsilyl was vulnerable to a 175 °C alkaline cement pore water solution with a pH of 10.74. Despite the degradation of hydrophobic trimethylsilyl groups, no alkali dissolution of silica aerogel was detected, as there were no noticeable bands related to amorphous or crystalline N-A-S and N-A-S-H phases in the region between 1000 to 870 cm^−1^, in agreement with the XRD results. In contrast, at 250 °C, anhydrous and hydrated N-A-S-(H) bands attributed to albite and hydrosodalite, respectively, were detected at 958 and 914 cm^−1^, indicating the alkali dissolution of silica aerogel at 250 °C. The band at 842 cm^−1^ was ascribed to CO_3_^2−^ in conjunction with the CO_3_^2−^ band at 853 cm^−1^.

**Figure 16:**
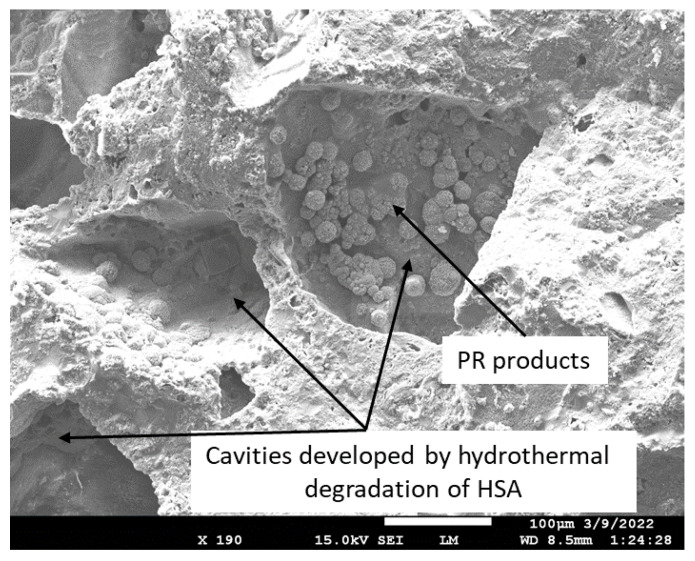
Photomicrograph of freshly fractured 250 °C-autoclaved Ca/HSA cement.

**Figure 17:**
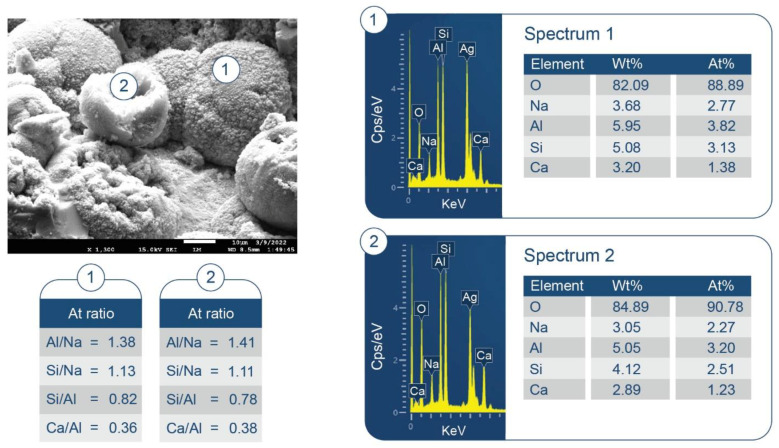
Photomicrograph of PR products of silica gel formed in a cavity of 250 °C-autoclaved Ca/HSA cement.

Furthermore, the information described above was supported by the SEM-EDX exploration of the microstructure in the 250 °C-autoclaved sample. The image of the fractured sample (Figure 16) showed a disappearance of all HSA aggregates due to their alkali dissolution and the formation of numerous large (~250 µm) cavities in their place. PR products of silica gel were observed in the cavities (Figure 17). EDX elemental analysis of PR products showed the presence of Na, Al, Si, and small amounts of Ca (less than 4%). The Al/Na, Al/Na, and Si/Al atomic ratios at locations No. 1 and 2 were attributable to hydrosodalite, possibly coexisting with some C,N-Al-S-H phases. However, the morphological features of these hydrosodalite crystals differed from the hydrosodalite formed in the CaP/FCS system, suggesting a poorly crystallized hydrosodalite.

#### 3.3.4. CaP/MGF System

**Figure 18:**
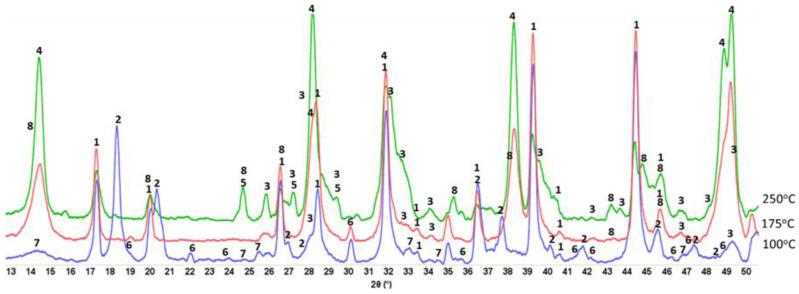
XRD patterns for 100-, 175-, and 250 °C-autoclaved CaP/MGF cements: 1-katoite (00-024-0217/01-077-0240/01-084-01354/01-074-9779); 2-gibbsite (01-076-1782); 3-hydroxyapatite (04-016-1647/04-011-6221/01-074-9779); 4-boehmite (04-010-5683); 5-calcium carbonate (01-085-6719); 6-krotite (00-023-1037); 7-grossite (00-023-1037); and 8-hydrosodalite (00-040-0102).

As seen in Figure 18, the XRD patterns of CaP/MGF cement (pore solution pH 11.05) at 100 and 175° were like those of the CaP/HSA system, namely, no crystalline anhydrous and hydrate N-A-S phases were detected. At 250 °C, unlike in the CaP/HSA system, only hydrosodalite formed as a minor phase, and no albite.

**Figure 19:**
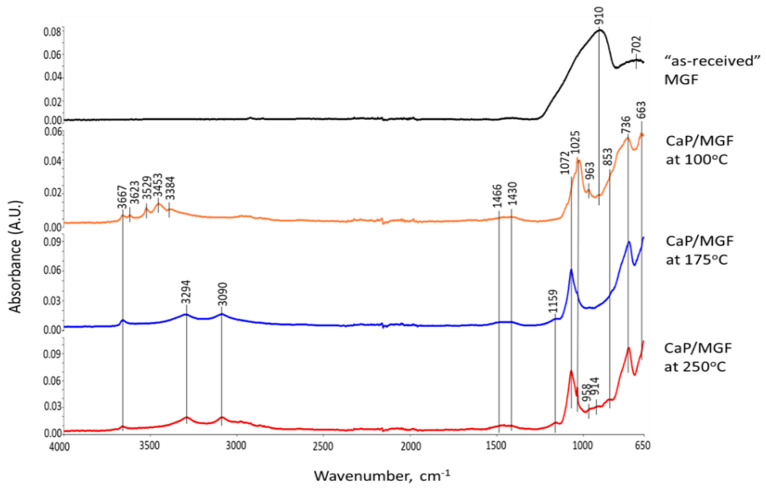
ATR-FTIR spectra for 100-, 175-, and 250 °C-autoclaved CaP/MGF cements.

Corresponding to XRD results, the N-A-S-H phases associated with hydrosodalite could be recognized from the bands at 958 and 914 cm^−1^ at 250 °C (Figure 19). As MGF is a non-crystalline calcium aluminosilicate (C-A-S), two bands at 910 and 702 cm^−1^ in the spectrum of “as-received” MGF were implicated in M-O-Si (M: Ca and Al) bonds. Therefore, the bands at 958 and 914 cm^−1^ may also belong to amorphous C,N-A-S-H phases formed by the PR of MGF with Na. Similar to HSA at 175 °C, no representative bands of N-A-S-H and C,N-A-S-H phases were found in the region of 1000–870 cm^−1^, verifying little susceptibility of MGF to PR at that temperature.

**Figure 20:**
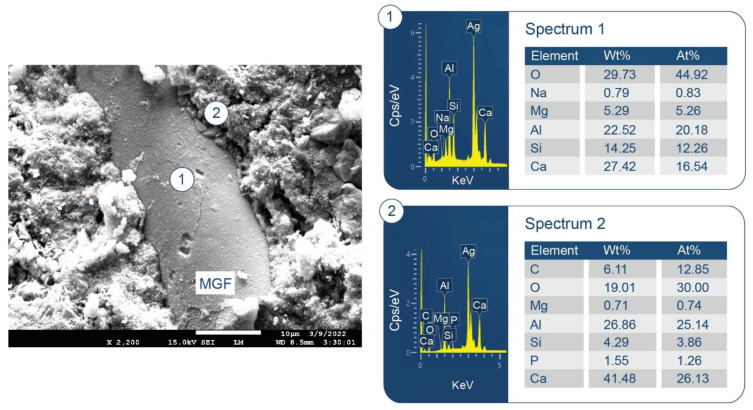
Photomicrograph of MGF of 250 °C-autoclaved Ca/MGF cement.

**Figure 21:**
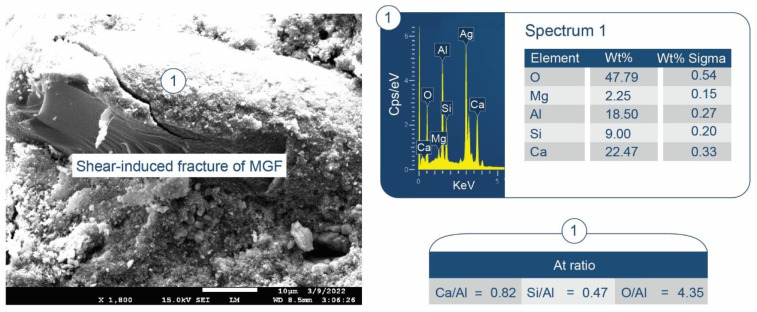
Photomicrograph of PR-changed MGF fiber of 250 °C-autoclaved Ca/MGF cement.

To support the above information, Figure 20 and Figure 21 display two different microstructures of MGF in CaP/MGF samples. Figure 20 shows the lack of PR of MGF, whereas Figure 21 demonstrates the degradation of MGF in PR. A SEM image of the non-reacted area of MGF presents a smooth fiber texture. The EDX of this smooth surface was consistent with the elemental composition of MGF, except for a small presence of Na. The EDX for location No. 2 of the cement adhering to MGF indicated the presence of three major elements (O, Al, and Ca) with atomic fractions ≥25% and three minor elements (Mg, Si, and P) with atomic fractions <4% (except C). Therefore, similar to the FCS surface, the cement was made by amorphous C-A-H and C-A-S-H with Ca/Al, Si/Al, and Ca/Si atomic ratios of 1.04, 0.15 and 6.77, respectively, verifying that the MGF surface was also susceptible to PR with alkaline calcium species. In contrast, the SEM image of the reacted area of MGF (Figure 21) highlighted the development of an excessive interfacial bonding between the cement and MGF, leading to a shear-induced fracture of MGF during sample preparation. The EDX determined that atomic fractions were similar in locations No. 1 and 2, with the only difference being a higher Si content in the PR-affected area, with the cement strongly adhering to the fiber. Thus, the atomic ratios of Si-rich amorphous C-A-S-H were as follows: Ca/Al = 0.82, Si/Al = 0.47, and Ca/Si = 1.75. On the other hand, it was very difficult to identify hydrosodalite phases on MGF surfaces. This phase probably formed in the interfacial cement–MGF region beneath the C-A-S-H layer.

#### 3.3.5. CaP/MCF System

**Figure 22:**
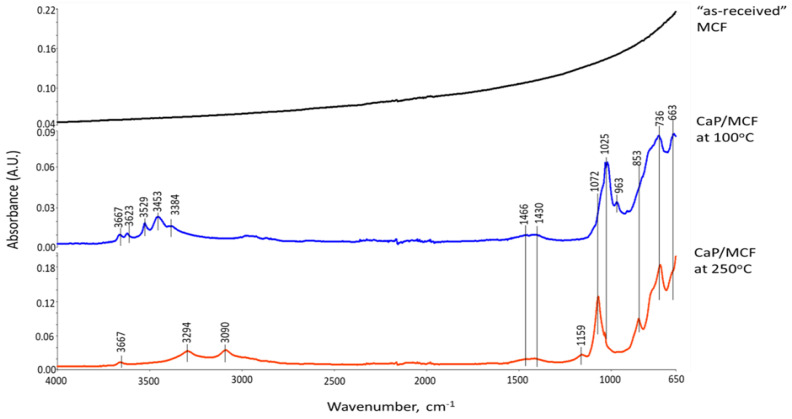
ATR-FTIR spectra for 100-, 175-, and 250 °C-autoclaved CaP/MCF cements.

Figure 22 presents ATR-FTIR spectra for 100-, 175-, and 250 °C-autoclaved CaP/MCF cements (pore solution pH 10.92). As expected, non-pozzolanic MCF had no affinity with Na^+^, Ca^2+^, and OH^−^ at 250 °C. In fact, the spectral features at 100 and 250 °C were the same as for neat CaP.

#### 3.3.6. Mechanic Properties

**Figure 23:**
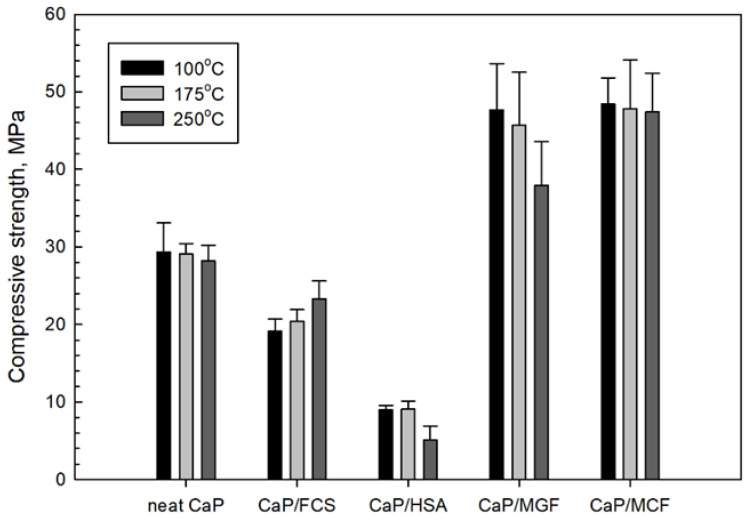
Compressive strengths of 100-, 175-, and 250 °C-autoclaved neat CaP and CaP cements made with different aggregates and reinforcements.

**Figure 24:**
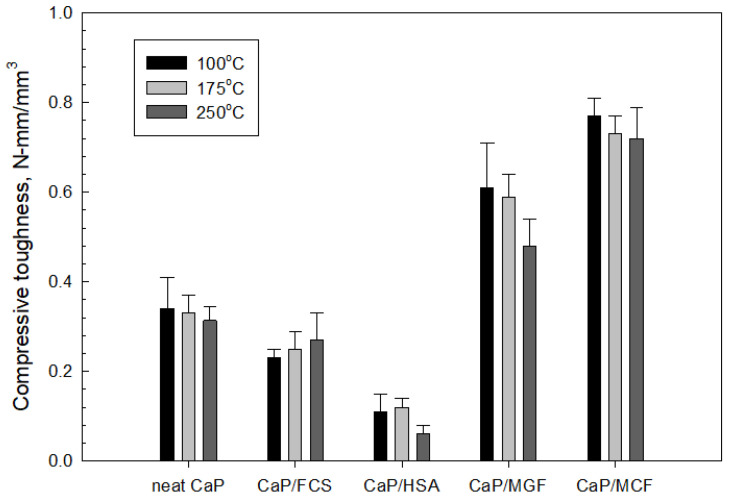
Compressive toughness of 100-, 175-, and 250 °C-autoclaved neat CaP and CaP cements made with different aggregates and reinforcements.

Figure 23 and Figure 24 give the compressive strength and compressive toughness for 100-, 175- and 250 °C-24 h-autoclaved CaP cements with and without aggregates and reinforcements. The neat CaP cement developed 29.3 MPa compressive strength after autoclaving at 100 °C. This strength tended to decline somewhat with increasing temperatures, to 29.1 MPa at 175 °C and 28.2 MPa at 250 °C. This slight loss in strength was due to two factors: changes in phase composition when hydroxyapatite (HOAp) became the major phase at 250 °C and the gibbsite to boehmite phase transition.

In contrast, upward trends could be seen for the CaP/FCS system with temperature increase. The compressive strength developed at 100 °C (9.1 MPa) increased at 175 °C (20.4 MPa) and increased further at 250 °C (23.3 MPa). This is a result of FCS shell pozzolanic activity at temperatures ≥175 °C. Pozzolanic reactions form N-A-S phases (crystallizing as albite) at 175 °C, and hydrosodalite and additional N-A-S-H phases at 250 °C. These PR products on the surface of FCS improved the adhesion of hard-shells to the CaP matrix, thereby resulting in increased compressive strength with the enhancement of pozzolanic activity at elevated temperatures. On the other hand, the strength of the CaP/HSA system at 100 °C dropped by nearly 43% to 5.1 MPa. It is evident that HSA itself did not contribute to compressive strength in CaP cement, and neither did the PR products of HSA. As described in the SEM-EDX study, the alkali dissolution of silica gel by the hydrothermal degradation of HSA at 250 °C engendered the creation of numerous cavities, including cavities as large as ~250 µm in size. This was the major reason why strength was drastically decreased. Furthermore, the contribution of HSA itself to the compressive strength of CaP was almost zero compared with that of FCS. Incorporation of MGF into CaP noticeably improved cement strength. The compressive strengths of 47.7 and 45.7 MPa developed at 100 and 175 °C was tantamount to nearly 1.6- and more than 5-times greater than the strengths of neat CaP and CaP/HSA, respectively, strongly demonstrating that MGF reinforcements could help recover the considerable strength loss brought about by adding HSA. However, at 250 °C, this strength markedly decreased, decreasing by 17% compared with its strength at 175 °C, implying that hydrosodalite formation from PR of MGF may have a negative effect in sustaining adequate compressive strength. This may be due to the conversion of ductile fibers into brittle ones by PR products. In contrast MCF, which is not susceptible to PR, showed a greater ability to recover strength loss due to the addition of HSA at temperatures up to 250 °C than MGF.

Compressive toughness depends on a balance between compressive strength and ductility. The data of compressive toughness mirrored the trends of compressive strength. MCF showed better performance than MGF. Importantly, MCF enhanced toughness, rather than compressive strength, for neat CaP. For example, compared with the ~1.6-fold improvement in compressive strength, toughness improved by ~2.2-fold. Nonetheless, both MGF and MCF offered improved toughness of the CaP cement matrix. The reinforcements can help considerably alleviate the losses in strength and toughness caused by HSA in CaP cement.

#### 3.3.7. Calorimetric Study of CaP Composites in Hydration

**Figure 25:**
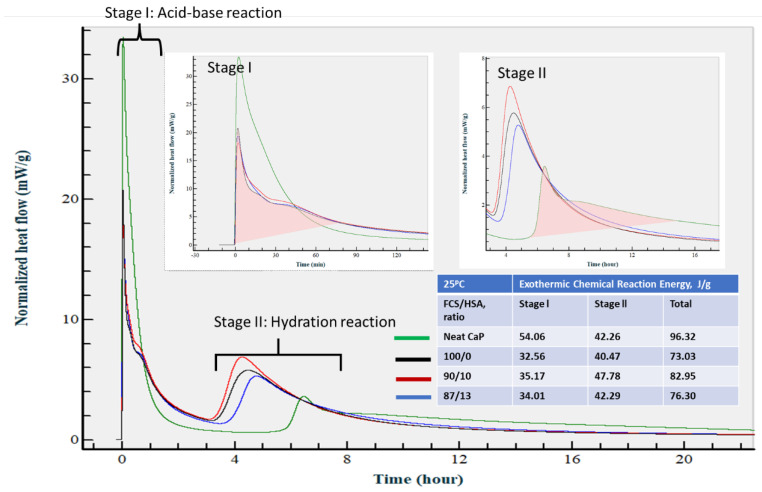
Calorimetric curves and normalized heat values for neat CaP cement and CaP composites made with FCS/HSA ratios of 100/0, 90/0, and 87/13 at temperature of 25 °C.

Figure 25 illustrates the isothermal calorimetry curves for neat CaP cement and CaP composite slurries with 100/0, 90/10, and 83/17 FCS/HSA ratios at 25 °C. There were two distinct heat release stages (I and II) during the hydration of the slurries. First stage (I) reactions occurred within the first ~80 min from the start of measurement; second stage (II) reactions took place between ~3 and ~15 h from the start of measurement. The total normalized heat released during the first stage, computed by integrating the area under the curve, was 56.06 J/g for neat CaP and 32.56, 35.17, and 34.01 J/g for 100/0, 90/10, and 83/17 CaP/HSA ratios, respectively. During stage II, the average total normalized heat value was 43.20 ± 2.75 J/g for all samples. The conspicuously high normalized heat value of neat CaP in stage I, compared with an average of 33.91 ± 1.07 J/g for the other CaP composites, can be explained by the acid–base reactions between orthophosphoric acid from SHMP and Ca^+^ and OH^−^ alkalis from CAC, as well as generally faster chemical reaction rates in neat CaP than in reactions of cement hydrate formations. stage II values were attributable to the hydration reactions of CAC, leading to the formation of katoite and gibbsite as end products. The average of total normalized heat release for all slurries was 82.15 ± 8.93 J/g. Although the contribution of CAC hydration to stage I heat release cannot be excluded completely, for the most part, CaP cement had two different cement-forming pathways induced by acid–base and hydration reactions. FCS pozzolanic reactions could have contributed to stage II heat release.

**Figure 26:**
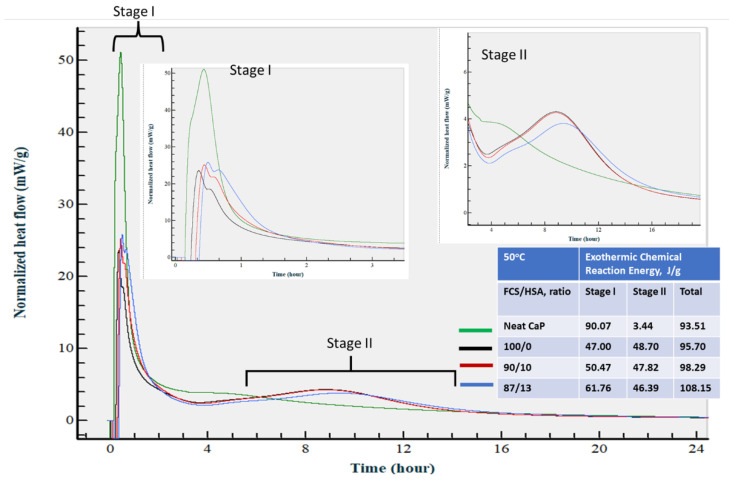
Calorimetric curves and normalized heat values for neat CaP cement and CaP composites made with FCS/HSA ratios of 100/0, 90/0, and 87/13 at temperature of 50 °C.

At an isothermal temperature of 50 °C (Figure 26), the calorimetry curve of neat CaP was characterized by a striking increase in normalized heat release during the first stage that extended to ~2 h from the start of measurement, and extremely low heat release during the second stage. This suggests that temperature increase accelerated CAC hydration reactions so that acid–base and hydration reactions happened during stage I, whereas cement hydration during stage II was minor. Increasing the temperature to 50 °C shortened the onset time of the neat cement hydration reactions from approximately 4 h 30 min at 25 °C to within 2 h. In contrast, heat release increased for all CaP/HSA composites during both stages, compared with those at 25 °C. Interestingly, the acid–base reaction heat release rate depended on the FCS/HSA ratios, namely, a decrease in the proportion of FCS to HSA enhanced heat release values. In fact, 61.76 J/g of an 87/13 ratio had 23.9 and 18.3% higher values than that of 100/0 and 90/10 ratios, respectively. As described in the CaP composite slurry properties, pH value increased with a decreasing FCS/HSA ratio. Thus, an 87/13 ratio with the highest pH of 10.58 increased the acid–base rection rate. An explanation for this could be that alkalinity was partially consumed by FCS during PR. This could have led to lower pH values in slurries with higher FCS content. The average value of total normalized heat release for all slurries was 98.91 ± 5.60 J/g.

**Figure 27:**
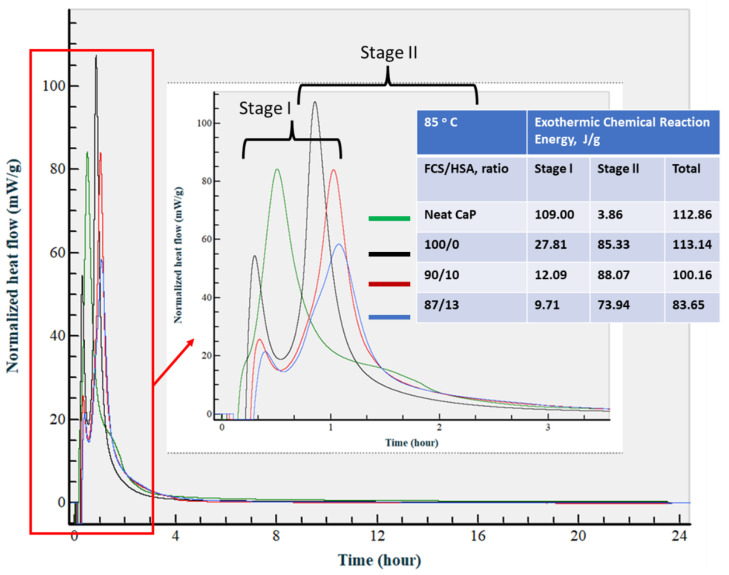
Calorimetric curves and normalized heat values for neat CaP cement and CaP composites made with FCS/HSA ratios of 100/0, 90/0, and 87/13 at temperature of 85 °C.

At 85 °C (Figure 27), there was a major shift in reaction onset time for stage II to an earlier time. As a result, both stages I and II started within the first 3 h after starting measurement. For neat CaP cement, the rate of the acid–base reaction during stage I further increased by 17.4% to 109 J/g, compared with that at 50 °C, whereas the heat release rate during stage II was similar to that at 50 °C. In contrast, for all composites, heat release during stage I markedly declined. Furthermore, this value showed a downward trend with decreasing FCS/HSA ratios. This phenomenon was reversed in comparison with results at 50 °C, whereas the heat release values during stage II considerably increased. For instance, the average value for all the composites, 82.45 ± 6.12 J/g, was 1.73 times greater than that at 50 °C. Moreover, stage I duration was ~3 h at 50 °C, but at 85 °C it was only ~45 min. The average of total heat release for all slurries was the highest in this series of experiments: 102.45 ± 12.06 J/g.

In this experiment, all the samples were prepared at 25 °C for 24 h prior to a steam pretreatment at 85 °C, following by an autoclave final treatment. Thus, the calorimetric results at 25 °C provided us with information on the two-stage chemical reaction pathway, with acid–base (first) and hydration (second) reactions. The first reaction occurred within the elapsed time of ~80 min from the start of measurement, whereas the second reaction happened between ~3 and ~15 h.

### 3.4. Compressive Strength and Toughness of 100 and 250 °C-Autoclaved CaP Cement Composites before and after Thermal Shock (TS) Tests

Next, we evaluated the thermal shock (TS) resistance of the MGF- and MCF-reinforced composites of CaP cement with various FCS/HSA ratios after two curing temperatures of 100 and 250 °C. The samples were subjected to three cycles of TS, with one cycle being 175 °C heat → 25 °C water quenching for 100 °C-autoclaved samples, and 250 °C → 25 °C water quenching for 250 °C-autoclaved samples.

**Figure 28:**
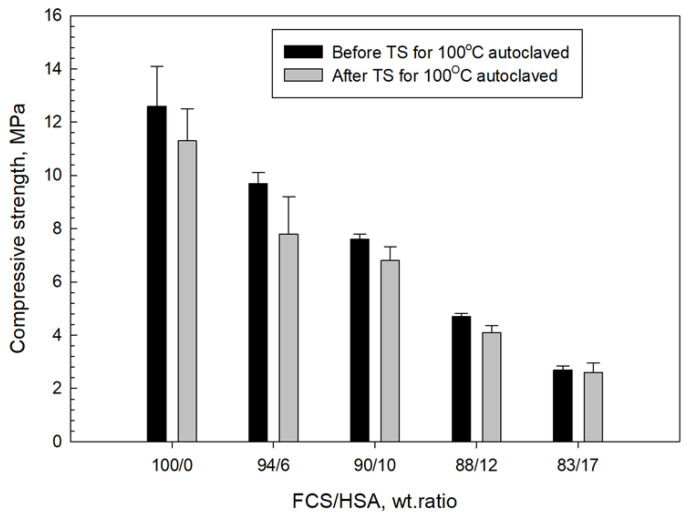
Compressive strength of 100 °C-autoclaved CaP cements made with different FCS/HSA ratios before and after thermal shock (TS) tests.

**Figure 29:**
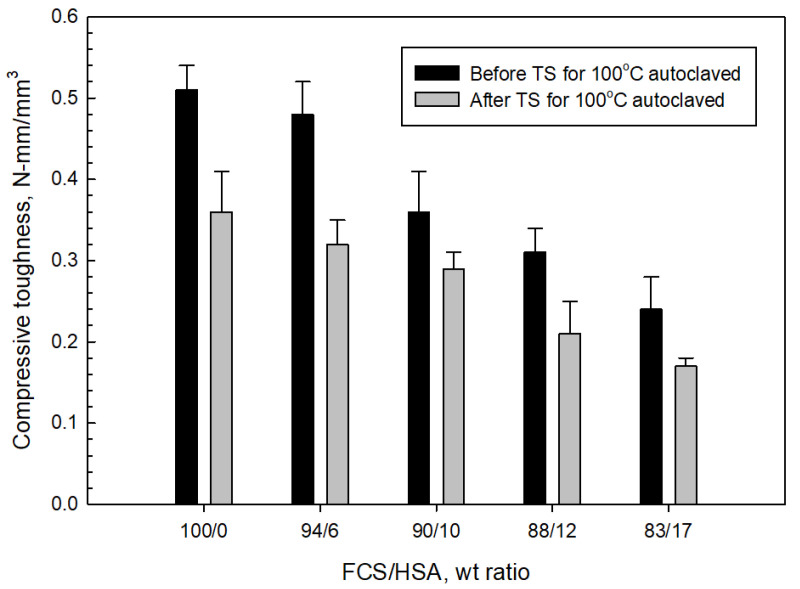
Compressive toughness of 100 °C-autoclaved CaP cements made with different FCS/HSA ratios before and after thermal shock (TS) tests.

Figure 28 and Figure 29 present the changes in compressive strength and toughness of 100 °C-autoclaved composites as a function of the FCS/HSA ratio for pre- and post-TS test samples. The pre-TS test sample with a 100/0 FCS/HSA ratio had a compressive strength of 12.6 MPa, compared with ~29 MPa in the neat CaP; this low strength was due to a much lower volumetric fraction of CAC in the composite systems. As evident from the figure, compressive strength tends to decline as the mass of HSA substituting FCS increases. The strength of the 87/13 ratio FCS/HSA was as low as 2.7 MPa, corresponding to a ~79% reduction compared with the 100/0 ratio composite. After TS tests, the 100/0 ratio composite exhibited a ~10% reduction in strength. This strength reduction was similar for composites with 94/6 and 90/10 ratios. In contrast, the 87/13 ratio composite showed the lowest reduction rate of ~4%. The average reduction for all samples including 100/0, 94/6, 90/10, 88/12, and 83/17 ratios was 13.30% ± 3.35.

For pre-TS samples, the toughness of 100/0 ratio was 0.51 N-mm/mm^3^, which was 33% higher than that of neat CaP, emphasizing a large improvement in toughness by MGF and MCF. Even the low-strength 87/13 ratio sample had a toughness of 0.24 N-mm/mm^3^, which was similar to that of the CaP/FCS system. After TS, the average toughness reduction in all samples was 28.61% ± 4.90, corresponding to ~2.2 times greater value than that of average compressive strength reduction. Despite a large reduction in toughness, the toughness values of 100/0, 96/4, and 90/10 ratios were greater than those for the CaP/FCS system. Furthermore, the toughness of 0.17 N-mm/mm^3^ for the 83/17 ratio sample was equivalent to a toughness improvement of ~55% compared with the CaP/HSA system, due to the reinforcements that minimized the considerable losses in toughness caused by the incorporation of HSA.

**Figure 30:**
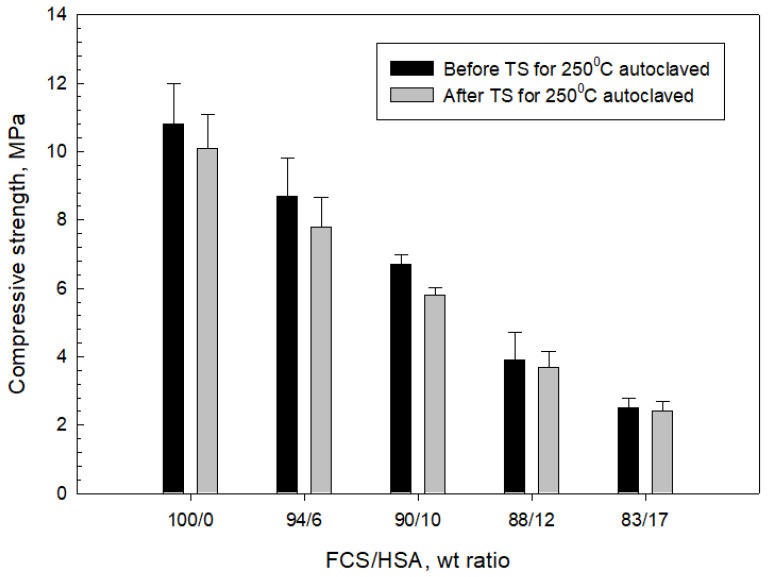
Compressive strength of 250 °C-autoclaved CaP cements made with different FCS/HSA ratios before and after thermal shock (TS) tests.

**Figure 31:**
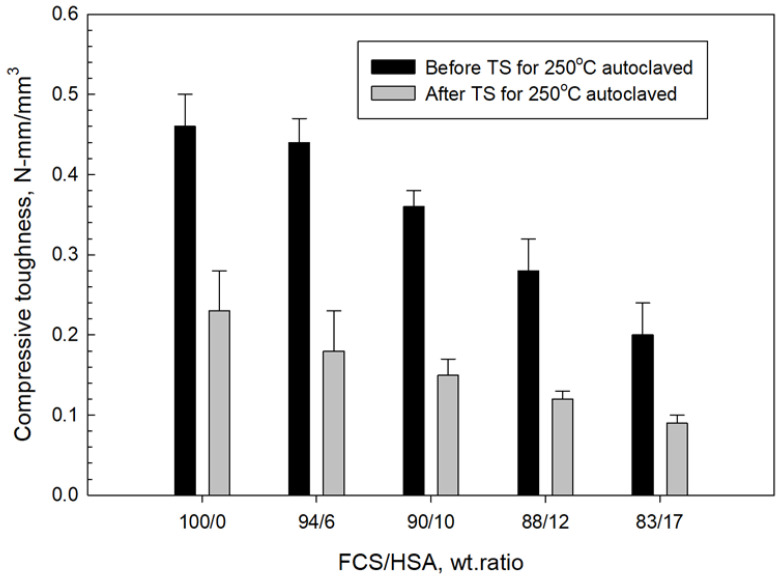
Compressive toughness of 250 °C-autoclaved CaP cements made with different FCS/HSA ratios before and after thermal shock (TS) tests.

Figure 30 and Figure 31 depict the changes in compressive strength and toughness of 250 °C-autoclaved CaP composites as a function of the FCS/HSA ratio for pre- and post-TS test samples. All pre-TS samples exhibited lower compressive strength compared with 100 °C-autoclaved ones.

The extent of strength reduction depended on the FCS/HSA ratios. As mentioned earlier, this strength decrease may be caused by two factors. One factor could be the phase transitions occurring in the cement, namely, HOAp becomes the major phase at 250 °C. The other is the gibbsite to boehmite phase transition. After a TS with the temperature gradient of ~225 °C, a small reduction (average 8.85% ± 2.9) in strength was observed, strongly suggesting that all composites possessed an adequate compressive strength for TS resistance. As for toughness, the pre-TS samples had a toughness similar to that of the 100 °C-autoclaved samples. This suggests that, regardless of the precipitation of N-A-S-H as a PR product of MGF at 250 °C, the MGF/MCF dual reinforcement aided in providing adequate toughness. However, TS for all samples engendered a striking reduction in toughness, with an average value of 56.14% ± 3.23, which was more than six times higher than the average reduction in compressive strength. As adequate toughness comes from a proper balance in compressive strength and ductility, this result suggests that the ductile nature of the lightweight composites was likely transformed to a brittle one at this TS temperature gradient. Thus, the development of a densified microstructure brought about by excessive PRs of FCS, HSA, and MGF in composites may cause brittleness at this TS heat temperature. For FCS, the excessive PR may lead to development of a strong, dense bond structure in cement/FCS shell interfacial boundary regions. For HSA, the PR may induce morphological alterations, such as the transformation of original nanoporous structure to a micro-porous one, whereas for MGF, the ductile fibers may be converted by PR into brittle ones. Although these composites demonstrated good strength resistance to TS, they did not sustain an adequate toughness following the TS tests.

### 3.5. Thermal Conductivity

**Figure 32:**
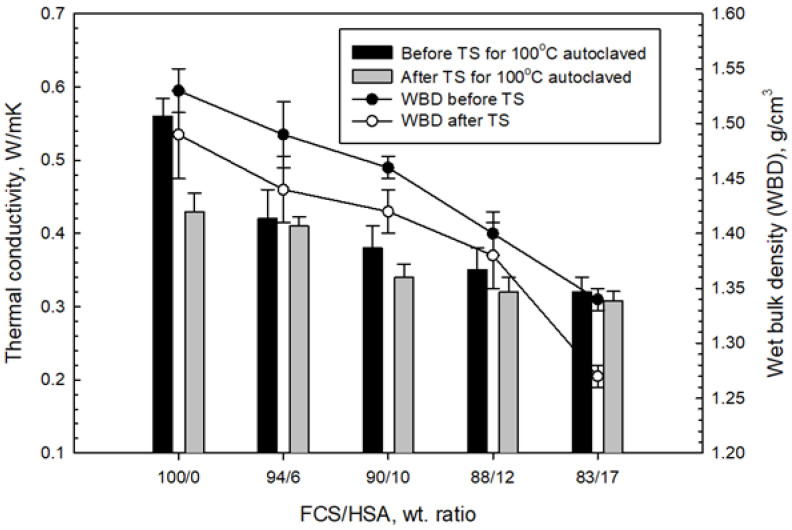
Thermal conductivity and wet bulk density of 100 °C-autoclaved CaP composites made with various FCS/HSA ratios before and after TS.

**Figure 33:**
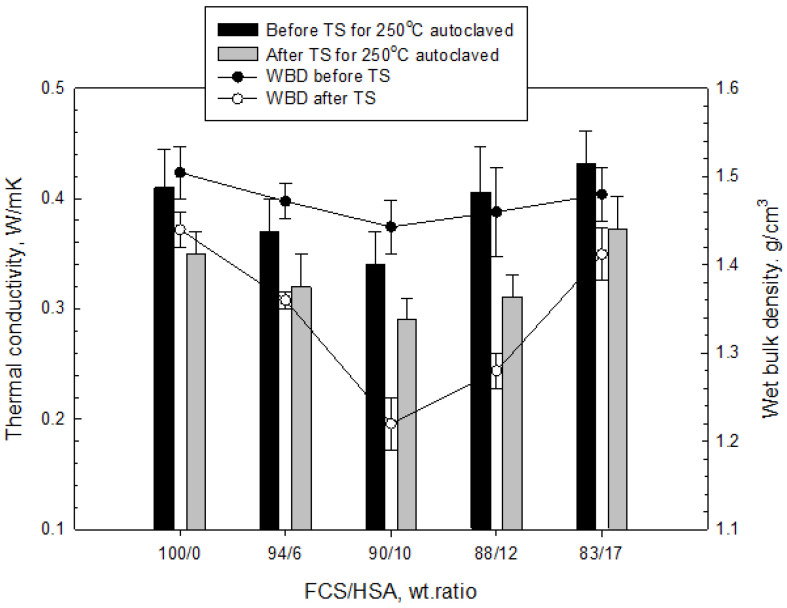
Thermal conductivity and wet bulk density of 250 °C-autoclaved CaP composites made with various FCS/HSA ratios before and after TS.

**Figure 34:**
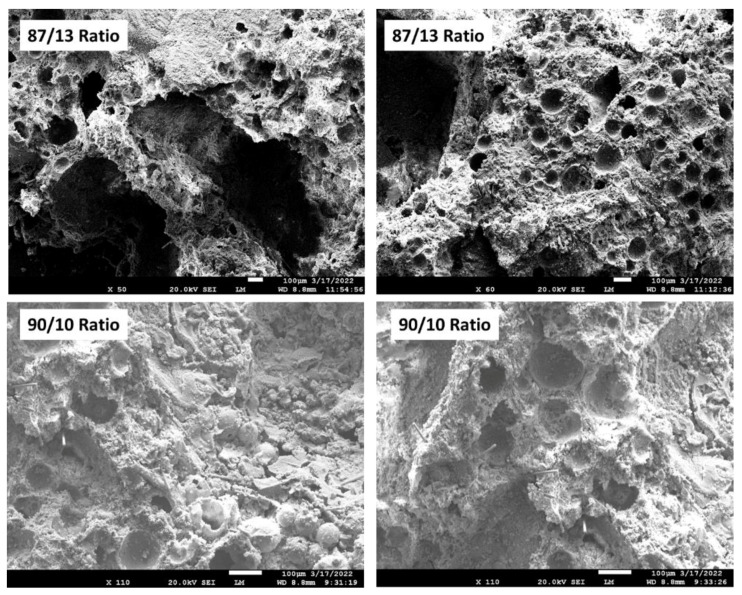
Microstructures of 90/10 and 87/13 samples, autoclaved at 250 °C.

Figure 32 and Figure 33 indicate the changes in thermal conductivity (TC) and water-saturated bulk density (WBD) as a function of the FCS/HSA ratio for 100- and 250 °C-autoclaved pre- and post-TS samples. For 100 °C-autoclaved pre-TS samples, the TC value decreased with the reduction of WBD. The 100/0 ratio sample had a TC of 0.56 W/mK with a WBD of 1.53 g/cm^3^. In contrast, the 83/17 ratio sample with the lowest WBD of 1.34 g/cm^3^ displayed a desirable decrease in the TC value by ~43% to 0.32 W/mK, due to the increased HSA content. Interestingly, all post-TS samples revealed decreases in both TC and WBD values. The 250 °C-autoclaved samples had two noticeable characteristics: (1) Further reduced TC and WBD values for 100/0, 94/6, and 90/10 ratios; (2) An upward trend of TC for 88/12 and 83/17 ratios. The 90/10 ratio sample had the lowest TC of 0.29 W/mK while exhibiting good TS resistance. The upward TC trend, on the other hand, resulted in 0.41 and 0.43 W/mK TC of the 88/12 and 83/17 ratios, respectively, which was tantamount to a ~21 and ~27% increase in TC. This implied that the incorporation of excessive HSA could lead to such undesirable results. The degradation of sample insulation performance was caused by the disintegration of HSA at 250 °C. As explained above, the high temperature degradation of HSA resulted in the development of microstructures with large pores. The loss of insulating air and water ingress through the porous structure led to an increased WBD. To support this, Figure 34 shows photomicrographs of 250 °C-autoclaved 90/10 and 83/17 ratio composites. The abundance of large cavities that have developed due to the decomposition of HSA is clearly visible in the 83/17 sample images, whereas the 90/10 sample shows few of these cavities. Small craters of ~100 µm size were formed by the debonding of FCS particles from the cement matrix. Such a hydrophilic porous structure allows water to permeate readily through the cement matrix, resulting in the increase of TC and WBD.

### 3.6. ATR-FTIR, XRD, and SEM-EDX Studies of CaP Cement Composites

**Figure 35:**
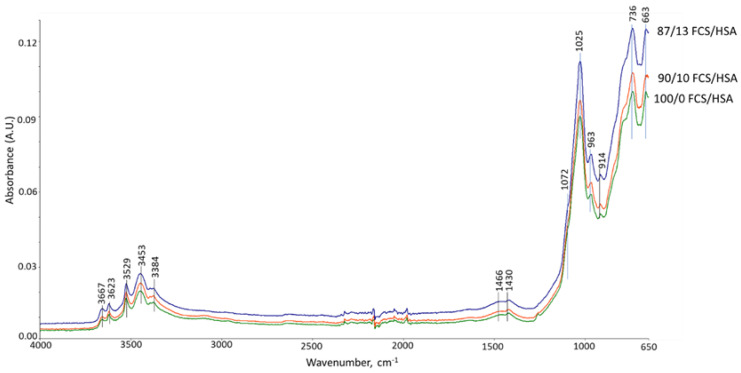
ATR-FTIR spectra of 100 °C-autoclaved 100/0, 90/10, and 87/13 ratio composites.

**Figure 36:**
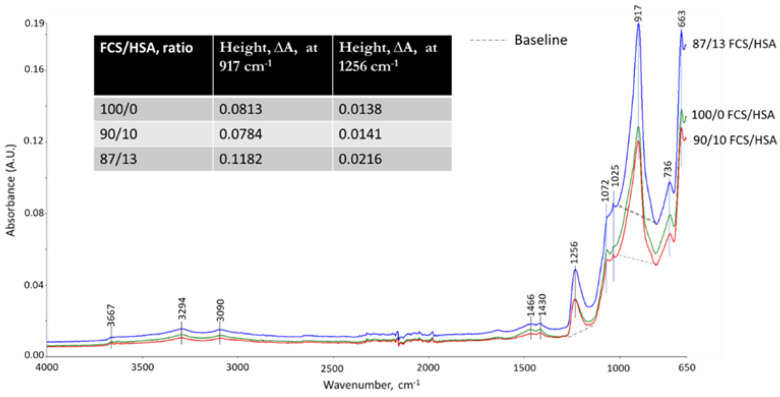
ATR-FTIR spectra of 250 °C-autoclaved 100/0, 90/10, and 87/13 ratio composites.

Figure 35 and Figure 36 show the ATR-FTIR spectra of 100- and 250 °C-autoclaved 100/0, 90/10, and 87/13 ratio samples. For all the samples autoclaved at 100 °C (Figure 35), the locations of all absorption bands, except for a weak band at 914 cm^−1^ ascribed to MGF, were similar to those of the neat CaP cement. In contrast, the spectra of 250 °C-autoclaved samples (Figure 36) showed emergence of two new bands at 1256 and 917 cm^−1^. The former band may be representing amorphous silica gel [91] from HSA. The peak intensity (height, ∆A) of this band for the 87/13 ratio sample was much greater than for the 100/0 and 90/10 ratio samples: 0.0216 ∆A of the 87/13 ratio was equivalent to a 54.3% increase from the averaged ∆A of the 100/0 and 90/10 ratios, indicating that a large free silica gel was liberated from the disintegrated HSA at 250 °C. As the 917 cm^−1^ band comes from amorphous and crystalline hydrated and anhydrous N-A-S(-H), C,N-A-S(-H), and C-A-S(-H), the striking increase of this band’s intensity for the 87/13 ratio sample clearly manifested from the precipitation of large amounts of these phases; the increase in this peak by 0.1182 ∆A was 47.8% bigger than the averaged ∆A of the 100/0 and 90/10 ratios. This correlated with another intense peak at 663 cm^−1^ attributed to these phases, as aforementioned in “CaP/FCS System”. Hence, PR of silica gels promoted the precipitation of these phases in the 87/13 ratio sample, but barely in the 100/0 and 90/10 samples. In other words, the pozzolanic activity of HSA in the 90/10 ratio sample was minor, similar to the case of FCS.

**Figure 37:**
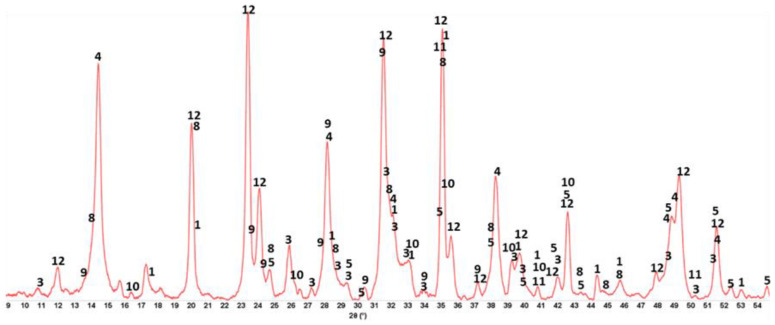
XRD pattern for 250 °C-autoclaved 87/13 ratio composite: 1-katoite (00-024-0217/01-077-0240/01-084-01354/01-074-9779); 3-hydroxyapatite (04-016-1647/04-011-6221/01-074-9779); 4-boehmite (04-010-5683); 5-calcium carbonate (01-085-6719); 8-hydrosodalite (00-040-0102); 9-albite (01-089-6427); 10-mullite (04-016-1586); 11-silicon oxide (00-014-0654); and 12-dmisteinbergite (04-011-6236).

To identify crystalline phases, XRD analysis was conducted. Figure 37 shows the XRD pattern of the 250 °C-autoclaved 87/13 ratio composite. The three PR-related crystalline phases included two anhydrous phases (albite (N-A-S), a Na-plagioclase feldspar and dmisteinbergite (CaAl_2_Si_2_O_8_, C-A-S), which is a high-temperature hexagonal polymorph of anorthite, a Ca-plagioclase feldspar) and one hydrous phase (hydrosodalite zeolite (N-A-S-H)). Among them, dmisteinbergite was the major PR product. Three other crystalline phases formed in CaP cement( katoite, HOAp, and boehmite) were also present as major phases.

**Figure 38:**
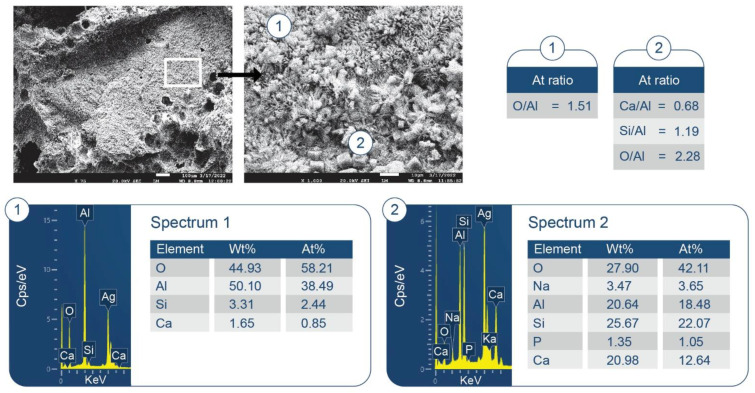
Photomicrographs of 250 °C-autoclaved 87/13 ratio composite at the location of disintegrated HSA.

**Figure 39:**
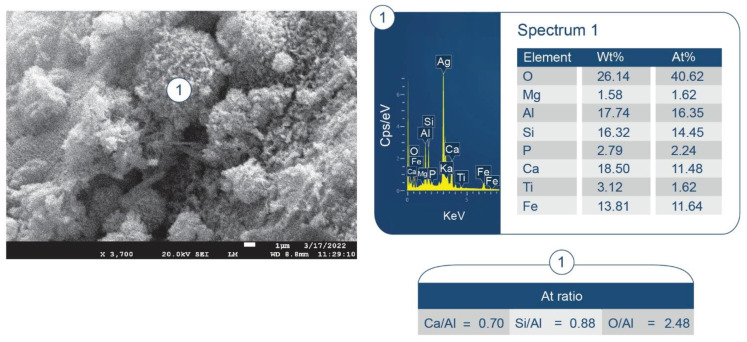
Photomicrograph of 250 °C-autoclaved 87/13 ratio composite at the location of FCS.

To support the above information, Figure 38 shows the photomicrograph and elemental composition of phases formed in the printing of disintegrated HSA on the freshly fractured surface of the 250 °C-autoclaved 87/13 ratio composite. The No. 1 location had two principal elements, O and Al, corresponding to an atomic O/A ratio of 1.51. This was likely a boehmite-rich region. The second location (No. 2) encompassed four major elements, O, Al, Si, and Ca, and the Ca/Al, Si/Al, and O/Al atomic ratios were 0.68, 1.19, and 2.28, respectively. These atomic ratios closely resemble those of dmisteinbergite: Ca/Al = 0.5, Si/Al = 1.0, and O/Al = 4.0. Interestingly, similar atomic ratios of Ca/Al = 0.70, Si/Al = 0.88, and O/Al = 2.48 were observed for the PR products formed on the surfaces of FCS shells where Ti and Fe signals came from the FCS itself (Figure 39). Nevertheless, the presence of mullite in the XRD patterns suggests limited pozzolanic activity in FCS. Thus, most of Si likely came from the disintegrated has, resulting in the precipitation of dmisteinbergite.

As in the case of albite, the orthosilicic acid (H_4_SiO_4_) derived from alkali (OH)^−^ dissolution of silica gels can serve as a source of Si for dmisteinbergite formation [83]:

Ca^2+^ + 2Al(OH)_4_^−^ + 2H_4_SiO_4 (*aq*)_ ↔ CaAl_2_Si_2_O_8_ + 8H_2_O.

The various crystalline phases formed in these composites may increase their TC (λ). For example, the λ of moist silica gel was reported to be in the range of ~0.19 W/mK at 0.1 water/gel wt. ratio to ~0.23 W/mK at 0.3 water/gel wt. ratio [92]. These λ values were considerably higher than the 0.012 W/mK of “as-received” HSA, corresponding to more than a fifteen-fold increase of the HSA. The thermal conductivity of ~3.3, ~2.0, and ~1.7 W/mK for N-A-S-H hydrosodalite [93], albite, and dmisteinbergite [94], respectively, was ~3.1-, ~1.9-, and ~1.6-fold higher than the ~1.1 W/mK thermal conductivity of hydroxyapatite [95] and ~1.05 W/mK of neat CAC hydrate containing C_3_AH_6_ and AH_3_ at a W/C ratio of 0.3 [96]. Nonetheless, these PR products may have not significantly affected composite TC. The boehmite that formed as a major phase of CaP cement at ≥175 °C has a very high λ of 30 W/mK. It is known as a high thermal conductor [97]. Thus, boehmite is an undesirable phase for thermally insulating cements. Nevertheless, in the case of the tested formulations, the products with high TC did not compromise the insulating nature of the composites

In summary, the incorporation of an excessive amount of HSA into the FCS/HSA hybrid insulating system engendered a negative effect on the thermal insulating performance of the 88/12 and 83/17 ratio composites, raising λ and WBD values compared with the 90/10 ratio samples at 250 °C.

## 4. Conclusions

Low TC and lightweight hydrophobic TS-resistant cement composites were formulated using a combination of FCS and HSA as insulating particles and CaP cement as a binder. The composites were cured at 100 or 250 °C and tested in three cycles of TS tests with temperature gradients of 150 or 225 °C (1 cycle: 175 °C heat for 24 h → 25 °C water quenching for 100 °C-autoclaved samples and 250 °C heat for 24 h → 25 °C water quenching for 250 °C autoclaved samples). Combining low-strength HSA with FCS and high-strength CaP cement binder resulted in an improvement in the mechanical properties of composites with very weak HSA particles and low TC values (below that of water) when measured under water saturated conditions. Further strength improvements were achieved with the use of reinforcement micro-glass and carbon fibers. As CaP cement has a lower pH (pH ~ 10 in tested blends) than OPC-based blends (pH ~ 13), this minimized pozzolanic reactions that lead to the degradation of solid FCS shells, release of insulating gasses, and the loss of cement integrity. The hydrophobic nature of HSA treated with HMDS provided cements with water-repellant properties, preventing water ingress and TC increase.

As a result, the optimized 90/10 FCS/HSA ratio samples after TS tests possessed a TC of 0.35 and 0.28 W/mK for 100 and 250 °C, respectively. However, at 250 °C, trimethylsilyl groups of HMDS underwent hydrothermal degradation, followed by trimethylsilyl-free silica aerogel alkali dissolution and development of a porous microstructure with large cavities in place of the dissolved HSA. This resulted in a 43% loss in strength compared with the 100- and 175 °C-made samples. Considering the hydrothermal degradation of the hydrophobic trimethylsilyl groups present on silica aerogel surfaces at 250 °C, the 90/10 FCS/HSA CaP composite has potential to be used as a thermally insulating, thermally shock-resistant well cement in mid-temperature range (~100 to 175 °C) geothermal energy storage systems. The compressive strength of more than 7 MPa for this lightweight composite persisted after three TS tests at 175 °C.

## Figures and Tables

**Table 1:** Oxide compositions of starting materials.

Component	Oxide Composition, wt%							
	Al_2_O_3_	CaO	SiO_2_	Fe_2_O_3_	Na_2_O	K_2_O	TiO_2_	MgO
CAC, #71	55.8	44.0	-	0.2	-	-	-	-
HSA	-	-	100	-	-	-	-	-
FCS	35.0	2.7	50.1	7.1	0.30	3.1	1.6	-
E-type MGF	11.4	28.6	55.0	0.9	0.6	-	0.7	2.8

**Table 2:** Properties of CaP cement slurries with different FCS/HSA ratios.

Property	FCS/HSA Weight Ratio				
	100/0	94/6	90/10	88/12	83/17
W/B ratio	0.53	0.49	0.5	0.54	0.55
Density, g/cm^3^	1.29	1.21	1.18	1.08	1.07
Slump, mm	78	82	83	80	79
pH	10.15	10.23	10.30	10.42	10.58

## Data Availability

The data presented in this study are available on request from the corresponding author.

## Abbreviations

CAC—calcium aluminate cement, FAF—fly ash, type F, FCS—fly ash cenospheres, HMDS—hexamethyldisilazane, HSA—hydrophobic silica aerogel, MCF—micro-carbon fiber, MGF—micro-glass fiber, PMHS—polymethylhydrosiloxane, PMS—polymethylsiloxane, PR—pozzolanic reactions, RIP—repolymerization-induced product, SMS—sodium metasilicate, TC—thermal conductivity, TMLR—total mass loss rate, TS—thermal shock, TSRC—thermal shock-resistant cement, W/B—water-to-blend ratio, WBD—water-saturated bulk density, WL—weight loss.

## References

[1] Sugama T., Pyatina T. (2021). Hydrophobic Lightweight Cement with Thermal Shock Resistance and Thermal Insulating Properties for Energy-Storage Geothermal Well Systems. Materials.

[2] Yu H., Li Q., Sun F. (2019). Numerical simulation of CO2 circulating in a retrofitted geothermal well. J. Pet. Sci. Eng..

[3] Lanahan M., Tabares-Velasco P.C. (2017). Seasonal Thermal-Energy Storage: A Critical Review on BTES Systems, Modeling, and System Design for Higher System Efficiency. Energies.

[4] Rao P., Momayez M., Runge K., Muralidharan K. (2020). Recent Developments in Thermally Insulating Materials Based on Geopolymers—A Review Article. Min. Metall. Explor..

[5] Kremieniewski M., Jasí B., Zima G., Kut Ł. (2021). Reduction of Fractionation of Lightweight Slurry to Geothermal Boreholes. Energies.

[6] Ignacio Velasco J., Traven K., Wisniewski W., Ducman V. (2022). Academic Editors: Ildiko Merta and Microstructural Characterization of Alkali-Activated Composites of Lightweight Aggregates (LWAs) Embedded in Alkali-Activated Foam (AAF) Matrices. Polymers.

[7] Shen L., Tan H., Ye Y., He W. (2022). Using Fumed Silica to Develop Thermal Insulation Cement for Medium–Low Temperature Geothermal Wells. Materials.

[8] Sliwa T., Ciepielowska M. Cement Slurries with Modified Thermal Conductivity for Geothermal Applications. Proceedings of the 47th Workshop on Geothermal Reservoir Engineering.

[9] US Department of Energy (2020). Energy Storage Grand Challenge Roadmap.

[10] Wendt D., Huang H., Zhu G., Kitz K., Green S., McLennan J., McTigue J., Neupane G. (2019). Flexible Geothermal Power Generation Utilizing Geologic Thermal Energy Storage.

[11] Wang K.S., Tseng C.J., Chiou I.J., Shih M.H. (2005). The thermal conductivity mechanism of sewage sludge ash lightweight materials. Cem. Concr. Res..

[12] Notaria B., Pinto J., Solorzano E., de Sajaa J.A., Dumont M., Rodrigues-Perez M.A. (2015). Experimental validation of the Knudsen effect in nanocellular polymeric foams. Polymer.

[13] Bhagat S., Kim Y.-H., Moon M.-J., Ahn Y.-S., Yeo J.-G. (2007). A cost-effective and fast synthesis of nanoporous SiO2 aerogel powders using water-glass via ambient pressure drying route. Solid State Sci..

[14] Hair M., Hertl W. (1971). Reaction of hexamethyldisilazane with silica. J. Phys. Chem..

[15] Gurav J., Jung I., Park H., Kang E., Nadargi D. (2010). Silica aerogel: Synthesis and applications. J. Nanomater..

[16] Julio M., Ilharco L. (2017). Ambient Pressure Hybrid Silica Monoliths with Hexamethyldisilazane_ From Vitreous Hydrophilic Xerogels to Superhydrophobic Aerogels _ Enhanced Reader.pdf. ACS Omega.

[17] Wagh P., Ingale S. (2002). Comparison of some physico-chemical properties of hydrophilic and hydrophobic silica aerogels. Ceram. Int..

[18] Koyano K., Tatsumi T., Yanaka Y., Nakata S. (1997). Stabilization of Mesoporous Molecular Sieves by Trimethylsilylation. J. Phys. Chem. B.

[19] Park D., Nishiyama N., Egashira Y., Ueyama K. (2001). Enhancement of Hydrothermal Stability and Hydrophobicity of a Silica MCM-48 Membrane by Silylation. Ind. Eng. Chem. Res..

[20] Castricum H., Sah A., Kreiter R., Blank D., Vente J., ten Elshof J. (2008). Hydrothermally stable molecular separation membranes from organically linked silica. J. Mater. Chem..

[21] Castricum H., Kreiter R., Veen H., Blank D., Vente J., ten Elshof J. (2008). High-performance hybrid pervaporation membranes with superior hydrothermal and acid stability. J. Membr. Sci..

[22] Jung J.S., Park H.C., Stevens R. (2001). Mullite ceramics derived from coal fly ash. J. Mater. Sci. Lett..

[23] Ignaszak Z., Baranowski A., Hycnar J., Zak M. (1990). Heat-Insulating, High-Temperature Materials on Cenosphere Base. Insulation Materials, Testing and Applications.

[24] Ranjbar N., Kuenzel C. (2017). Cenospheres: A review. Fuel.

[25] Data Sheet of EconoStar 500 Cenospheres. https://www.cenostar.com/pages/cenospheres.

[26] Ichikawa T., Miura M. (2007). MOdified model of alakli-silica reaction. Cem. Concr. Res..

[27] Mindess S., Hewlett P., Liska M. (2019). Resistance of Concrete to Destructive Agencies. Lea’s Chemistry of Cement and Concrete.

[28] Wang J.Y., Zhang M.H., Li W., Chia K.S., Liew R.J.Y. (2012). Stability of cenospheres in lightweight cement composites in terms of alkali-silica reaction. Cem. Concr. Res..

[29] Manders P., Bader M. (1981). The strength of hybrid glass/carbon fibre composites. J. Mater. Sci..

[30] Sudarisman R., de San Miguel B., Davies I. The effect of partial substitution of E-glass fibre for carbon fibre on the mechanical properties of CFRP composites. Proceedings of the International Conference on Materials and Metallurgical Technology, ICONNET 2009.

[31] Dong C., Ranaweera-Jayawardena H., Davies I. (2012). Flexural properties of hybrid composites reinforced by S-2 glass and T700S carbon fibres. Compos. Part B Eng..

[32] Hung P., Lau K., Cheng L., Leng J., Hui D. (2018). Impact response of hybrid carbon/glass fibre reinforced polymer composites designed for engineering applications. Compos. Part B Eng..

[33] McIvor S., Darby M., Wostenholm G., Yates B., Banfield L., King R., Webb A. (1990). Thermal conductivity measurements of some glass fibre- and carbon fibre-reinforced plastics. J. Mater. Sci..

[34] Cao X., Liu J., Li Q., Hu E., Fan F. (2015). Study of the thermal insulation properties of the glass fiber board used for interior building envelope.pdf. Energy Build..

[35] Purnell P., Short N., Page C., Majumdar A. (2000). Microstructural observations in new matrix glass fibre reinforced cement. Cem. Concr. Res..

[36] He P., Zhang B., Lu J., Poon C. (2021). Reaction mechanisms of alkali-activated glass powder-ggbs-CAC composites. Cem. Concr. Compos..

[37] Ranjbar N., Zhang M. (2020). Fiber-reinforced geopolymer composites: A review. Cem. Concr. Compos..

[38] Teixeira Marvila M., Rangel Garcez De Azevedo A., De Matos P.R., Monteiro S.N., Maurício C., Vieira F., Zichella L. (2021). Materials for Production of High and Ultra-High Performance Concrete: Review and Perspective of Possible Novel Materials. Materials.

[39] Barluenga G. (2010). Fiber-matrix interaction at early ages of concrete with short fibers. Cem. Concr. Res..

[40] Butler M., Hempel S., Mechtcherine V. (2011). Modelling of ageing effects on crack-bridging behavior of AR-glass multifilament yarns embedded in cement-based matrix. Cem. Concr. Res..

[41] Kartal A., Erkey C. (2010). Surface Modification of Silica Aerogels by Hexamethyldisilazane carbondioxide mixtures and their phase behavior. J. Supercrit. Fluids.

[42] Gun’ko V., Vedamuthu M., Henderson G., Blitz J. (2000). Mechanism and Kinetics of Hexamethyldisilazane Reaction with a Fumed Silica Surface. J. Colloid Interface Sci..

[43] Slavov S., Sanger A., Chuang K. (2000). Mechanism of Silation of Silica with Hexamethyldisilazane. J. Phys. Chem. B.

[44] Haukka S., Root A. (1994). The reaction of hexamethyldisilazane and subsequent oxidation of trimethylsilyl groups on silica studied by solid-state NMR and FTIR. J. Phys. Chem..

[45] Nagappan S., Ha C.-S. (2014). Effect of Sodium Hydroxide on the Fast Synthesis of Superhydro-phobic Powder from Polymethylhydrosiloxane. J. Coat. Sci. Technol..

[46] Groza A., Surmeian A. (2015). Characterization of the oxides present in a polydimethylsiloxane layer obtained by polymerisation of its liquid precursor in corona discharge. J. Nanomater..

[47] Warring S., Beattie D., McQuillan J. (2016). Surficial Siloxane-to-Silanol Interconversion during Room-Temperature Hydration/Dehydration of Amorphous Silica Films Observed by ATR-IR and TIR-Raman Spectroscopy. Langmuir.

[48] Efimov A.M., Pogareva V.G., Shashkin A.V. (2003). Water-related bands in the IR absorption spectra of silicate glasses. J. Non-Cryst. Solids.

[49] Cheng F., Cao Q., Guan Y., Cheng H., Wang X., Miller J. (2013). FTIR analysis of water structure and its influence on the flotation of arcanite (K_2_SO_4_) and epsomite (MgSO_4_·7H_2_O). Int. J. Miner. Process..

[50] Iacob C., Sangoro J., Papadopoulos P., Schubert T., Naumov S., Valiullin R., Karger J., Kremer F. (2010). Charge transport and diffusion of ionic liquids in nanoporous silica membranes. Phys. Chem. Chem. Phys..

[51] Kytokivi A., Haukka S. (1997). Reactions of HMDS, TiCl4, ZrCl4, and AlCl3 with Silica As Interpreted from Low-Frequency Diffuse Reflectance Infrared Spectra. J. Phys. Chem. B.

[52] Flores-Vivian I., Hejazi V., Kozhukhova M.I., Nosonovsky M., Sobolev K. (2013). Self-assembling particle-siloxane coatings for superhydrophobic concrete. ACS Appl. Mater. Interfaces.

[53] Camino G., Lomakin S.M., Lazzari M. (2001). Polymethyl silozane thermal degradation: Part I. Kinetic aspects. Polymer.

[54] Singh U.B., Gupta S.C., Flerchinger G.N., Moncrief J.F., Lehmann R.G., Fendinger N.J., Traina S.J., Logan T.J. (2000). Modeling polydimethylsiloxane degradation based on soil water content. Environ. Sci. Technol..

[55] Purkayastha A., Baruah J.B. (2004). Synthetic methodologies in siloxanes. Appl. Organomet. Chem..

[56] Varaprath S., Stutts D.H., Kozerski G.E. (2006). A primer on the analytical aspects of silicones at trace levels-challenges and artifacts-A review. Silicon Chem..

[57] Chainet F., Meur L.L., Lienemann C.P., Ponthus J., Courtiade M., Donard O.F.X. (2013). Characterization of silicon species issued from PDMS degradation under thermal cracking of hydrocarbons: Part 1-Gas samples analysis by gas chromatography-time of flight mass spectrometry. Fuel.

[58] Ducom G., Laubie B., Ohannessian A., Germain P., Chatain V. (2013). Hydrolysis of polydimethylsiloxane fluids in controlled aqueous solutions. Water Sci. Technol..

[59] Perez M., Vazquez T., Trivino F. (1983). Study of stabilized phases in high alumina cement mortars Part I Hydration at elevated temperatures followed by carbonation. Cem. Concr. Res..

[60] Klaus S., Neubauer J., Goetz-Neuhoeffer F. (2013). Hydration kinetics of CA2 and CA—Investigations performed on a synthetic calcium aluminate cement. Cem. Concr. Res..

[61] Reardon E. (1990). An ion interaction model for the determination of chemical equilibria in cement/water systems. Cem. Concr. Res..

[62] Corbridge D., Lowe E. (1954). The infra-red spectra of inorganic phosphorus compounds. Part II. Some salts of phosphorus oxy-acids. J. Chem. Soc..

[63] Sugama T., Weber L., Brother L. (2000). Sodium-polyphosphate-modified fly ash/calcium aluminate blend cement: Durability in wet, harsh geothermal environments. Mater. Lett..

[64] Moustafa Y., El-Egili K. (1998). Infrared spectra of sodium phosphate glasses. J. Non-Cryst. Solids.

[65] Garcia-Lodeiro I., Irisawa K., Jin F., Meguro Y., Kinoshita H. (2018). Reduction of water content in calcium aluminate cement with/out phosphate modification for alternative cementation technique. Cem. Concr. Res..

[66] Galameau E., Gehr R. (1997). Phosphorus removal from wastewaters: Experimental and theoretical support for alternative mechanisms. Water Res..

[67] Holm T., Edwards M. (2003). Metaphosphate reversion in Laboratory and Pipe-Rig Experiments. J. Am. Water Work Assoc..

[68] Georgantas D., Grigoropoulou H. (2007). Orthophosphate and metaphosphate ion removal from aqueous solution using alum and aluminum hydroxide. J. Colloid Interface Sci..

[69] Lu J., Sun M., Yuan Z., Qi S., Tong Z., Li L., Meng Q. (2019). Innovative insight for sodium hexametaphosphate interaction with serpentine. Colloinds Surf. A Physicochem. Eng. Asp..

[70] Glonek T. (2021). Did Cyclic Metaphosphates Have a Role in the Origin of Life?. Orig. Life Evol. Biosph..

[71] Tarte P. (1967). Infra-red spectra of inorganic aluminates and characteristic vibrational frequencies of AlO4 tetrahedra and AlO6 octahedra. Spectrochim. Acta Part A Mol. Spectrosc..

[72] Fernandez-Carrasco L., Torrens-Martin D., Morales L.M., Martinez-Ramirez S., Theophile T. (2012). Infrared spectroscopy in the analysis of building and construction materials. Infrared Spectroscopy-Materials Science, Engineering and Technology.

[73] Xyla A.G., Koutsoukos P.G. (1989). Quantitative Analysis of Calcium Carbonate Polymorphs by Infrared Spectroscopy. J. Chem. Soc. Faraday Trans. 1 Phys. Chem. Condens. Phases.

[74] Ylmen R., Jaglid U. (2013). Carbonation of Portland cement studied by diffuse reflection Fourier transform infrared spectroscopy. Int. J. Concr. Struct. Mater..

[75] Rehman I., Bonfield W. (1997). Characterization of hydroxyapatite and carbonated apatite by photo acoustic FTIR spectroscopy. J. Mater. Sci. Mater. Med..

[76] Berzina-Cimdina L., Borodajenko N., Theophanides T. (2012). Research of Calcium Phosphates Using Fourier Transform Infrared Spectroscopy. Infrared Spectroscopy-Materials Science, Engineering and Technology.

[77] Fujita S., Suzuki K., Shibasaki Y. (2002). The mild hydrothermal synthesis of hydrogrossular from coal ash. J. Mater. Cycles Waste Manag. Vol..

[78] Kolesov B., Geiger C. (2005). The vibrational spectrum of synthetic hydrogrossular (katoite) Ca3Al2(O4H4)3: A low-temperature IR and Raman spectroscopic study. Am. Mineral..

[79] Eisinas A., Dambrauskas T., Baltakys K., Ruginyte K. (2019). The peculiarities of mayenite formation from synthetic katoite and calcium monocarboaluminate samples in temperature range 25–1150 °C. J. Therm. Anal. Calorim..

[80] Kiss A.B., Keresztury G., Farkas L. (1980). Raman and ir spectra and structure of boehmite (γ-AlOOH). Evidence for the recently discarded D 17 2h space group. Spectrochim. Acta Part A Mol. Spectrosc..

[81] Morterra C., Emanuel C. (1992). Infrared Study of some Surface Properties of Boehmite (y-AlO2H). J. Chem. Soc. Faraday.

[82] Boumaza A., Favaro L., Lédion J., Sattonnay G., Brubach J.B., Berthet P., Huntz A.M., Roy P., Tétot R. (2009). Transition alumina phases induced by heat treatment of boehmite: An X-ray diffraction and infrared spectroscopy study. J. Solid State Chem..

[83] Elakneswaran Y., Ubaidah A., Takeya M., Shimokawara M., Okano H. (2021). Effect of Electrokinetics and Thermodynamic Equilibrium on LowSalinity Water Flooding for Enhanced Oil Recovery in Sandstone Reservoirs. ACS Omega.

[84] Bertoluzza A., Fagnano C., Antonietta Morelli M., Gottardi V., Guglielmi M. (1982). Raman and infrared spectra on silica gel evolving toward glass. J. Non-Cryst. Solids.

[85] Padmaja P., Anilkumar G.M., Mukundan P., Aruldhas G., Warrier K.G.K. (2001). Characterisation of stoichiometric sol-gel mullite by fourier transform infrared spectroscopy. Int. J. Inorg. Mater..

[86] Saikia B.J., Parthasarathy G., Sarmah N.C. (2008). Fourier transform infrared spectroscopie etimation of crystallinity in SiO2 based rocks. Bull. Mater. Sci..

[87] Guan W., Ji F., Fang Z., Fang D., Cheng Y., Yan P., Chen Q. (2014). Low hydrothermal temperature synthesis of porous calcium silicate hydrate with enhanced reactivity SiO2. Ceram. Int..

[88] Urhan S. (1987). Alkali silica and pozzolanic reactions in concrete. Part 1: Interpretation of published results and an hypothesis concerning the mechanism. Cem. Concr. Res..

[89] Mertens G., Snellings R., Van Balen K., Bicer-Simsir B., Verlooy P., Elsen J. (2009). Pozzolanic reactions of commn natural zeolites with lime and parameters affecting their reactivity. Cem. Concr. Res..

[90] Fernandez R., Martirena F., Scrivener K.L. (2011). The origin of the pozzolanic activity of calcined clay minerals: A comparison between kaolinite, illite and montmorillonite. Cem. Concr. Res..

[91] Almeida R., Pantano C. (1990). Structural investigation of silica gel films by infrared spectroscopy. J. Appl. Phys..

[92] Henrik B., Ernest K., Bo C. (1984). Thermal conductivity of a microporous particulate medium: Moist silica gel. Int. J. Heat Mass Transf..

[93] Griesinger A., Spindler K., Hahne E. (1999). Measurements and theoretical modelling of the effective thermal conductivity of zeolites. Int. J. Heat Mass Transf..

[94] Linvill M.L., Vandersande J.W., Pohl R.O. (1984). Thermal conductivity of feldspars. Bull. Minéral..

[95] Bento A.C., Almond D.P., Brown S.R., Turner I.G. (1996). Thermal and optical characterization of the calcium phosphate biomaterial hydroxyapatite. J. Appl. Phys..

[96] Ukrainczyk N., Matusinović T. (2010). Thermal properties of hydrating calcium aluminate cement pastes. Cem. Concr. Res..

[97] Duwe S., Arlt C., Aranda S., Riedel U., Ziegmann G. (2012). A detailed thermal analysis of nanocomposites filled with SiO 2, AlN or boehmite at varied contents and a review of selected rules of mixture. Compos. Sci. Technol..

